# Template-based intervention in Boolean network models of biological systems

**DOI:** 10.1186/s13637-014-0011-4

**Published:** 2014-07-19

**Authors:** Michael P Verdicchio, Seungchan Kim

**Affiliations:** 1 Department of Mathematics and Computer Science, The Citadel, Charleston, 29409 SC USA; 2 Integrated Cancer Genomics Division, Translational Genomics Research Institute, Phoenix, 85004 AZ USA

**Keywords:** Boolean networks, Attractors, Logic minimization, Intervention, Leukemia

## Abstract

**Motivation:**

A grand challenge in the modeling of biological systems is the identification of key variables which can act as targets for intervention. Boolean networks are among the simplest of models, yet they have been shown to adequately model many of the complex dynamics of biological systems. In our recent work, we utilized a logic minimization approach to identify quality single variable targets for intervention from the state space of a Boolean network. However, as the number of variables in a network increases, the more likely it is that a successful intervention strategy will require multiple variables. Thus, for larger networks, such an approach is required in order to identify more complex intervention strategies while working within the limited view of the network’s state space. Specifically, we address three primary challenges for the large network arena: the first challenge is how to consider many subsets of variables, the second is to design clear methods and measures to identify the best targets for intervention in a systematic way, and the third is to work with an intractable state space through sampling.

**Results:**

We introduce a multiple variable intervention target called a template and show through simulation studies of random networks that these templates are able to identify top intervention targets in increasingly large Boolean networks. We first show that, when other methods show drastic loss in performance, template methods show no significant performance loss between fully explored and partially sampled Boolean state spaces. We also show that, when other methods show a complete inability to produce viable intervention targets in sampled Boolean state spaces, template methods maintain significantly consistent success rates even as state space sizes increase exponentially with larger networks. Finally, we show the utility of the template approach on a real-world Boolean network modeling T-LGL leukemia.

**Conclusions:**

Overall, these results demonstrate how template-based approaches now effectively take over for our previous single variable approaches and produce quality intervention targets in larger networks requiring sampled state spaces.

**Electronic supplementary material:**

The online version of this article (doi:10.1186/s13637-014-0011-4) contains supplementary material, which is available to authorized users.

## Introduction

### Motivation

The very nature of medicine is to know when and how to intervene in order to shift the steady behavior of a system to a more desirable state [1]. Ideally, such interventions would be as minimally damaging as possible; however, we know that especially with diseases such as cancer, interventions like chemotherapy are anything but minimal. In the path towards personalized medicine and individualized treatments with minimal collateral damage, designing and studying interventions that take advantage of our system-level understanding and available data is and will remain of paramount importance, as working with computational models allows us to perform tests, execute simulations, and make predictions in inexpensive ways that require no human subjects [2].

Biological systems are complex in many dimensions as endless transportation and communication networks all function simultaneously [3]. Despite its simplicity, the Boolean network model has proven to be quite viable at approximating certain aspects of biological processes [1]. For example, it has been used to simulate the yeast cell cycle [4], which we looked at closely in our work [5]. It has also been used to simulate the expression pattern of segment polarity genes in *Drosophila melanogaster*[6], as well as the vocal communication system of the songbird brain [7],[8]. Since Kauffman’s seminal work [9], there have been countless variations and extensions of the use of Boolean networks for modeling biological systems, and various inference procedures have been proposed for them [10]–[12].

An intervention, in the context of a Boolean network, is defined as a modification (set/reset) to one or more variables in an attractor state of a source basin with the intention that network rules will transition to any state in a given goal basin (thus eventually reaching the attractor of the goal basin). In our recent work [5], we employed a logic reduction algorithm to reduce the Boolean states comprising the basins of attraction to minimal representations, and from those minimizations, we identified high-quality intervention targets comprised of single variables. However, as the number of variables in a biological network increases, the more likely it is that a successful intervention target will require the combined efforts of multiple variables. Thus, for larger networks, a new approach is required beyond our previous work in order to identify coherent, multi-valued intervention targets while working in with the limited view of the network’s state space.

### Related work

In this section, we detail pioneering efforts in the Boolean network field, especially in its application to biology, and we describe other attempts to identify key variables in networks while dealing with increasingly large state spaces. In the end, we find a remaining need for the results presented in this study.

Within the world of *in silico* modeling and intervention studies, significant groundwork has been laid. Boolean networks allow modeling at the most simplified extreme of the spectrum due to their coarse discretization of values to 0 and 1 and their simplified, rule-based update mechanism, yet have still been shown to adequately model complex behaviors seen in the biological system. In the next section, we give formal descriptions of Boolean networks and the basin of attraction field they generate. Over 30 years after Kauffman’s seminal work [9], Shmulevich et al. [13] pioneered work on a stochastic extension to the model called probabilistic Boolean networks (PBNs), which share the rule-based nature of Boolean networks but also handle uncertainty well. Within this extended framework of PBNs, studies were performed by Datta et al. [14],[15], which focused on external system control; studies by Pal et al. [16] and Choudhary et al. [17] explored intervention in PBNs to avoid undesirable states. Our previous work [18] mapped the biological intervention planning problem to a finite horizon partially observable Markov decision process (POMDP). While this formulation generates high-quality sequentially administered intervention plans, it takes as input a set of variables upon which to intervene and is not designed to elucidate the intervention targets themselves.

One major challenge in using Boolean networks is the exponential growth of the basin of attraction field, or state transition diagram (described below), with the linear growth of the number of variables, prompting others to work in the Boolean framework itself to achieve some kind of improvement. The approach of Richardson [19] attempted to shrink the size of the state space through the careful removal of ‘frozen nodes’ and network leaf nodes. The smaller state space then lent itself more readily to the discovery of attractors and basins by sampling methods. Dubrova et al. [20] explored properties of random Boolean networks, particularly their robustness in the face of topological changes and the removal of ‘redundant vertices’, thus shrinking the state space. Saadatpour et al. [21] build on the work of Naldi et al. [22] with a method of network simplification which eliminates stabilized nodes and mediator nodes, which can reduce networks to just a handful of significant variables. In fact, we apply their strategy later in this work to slightly reduce a network from 60 to 43 variables. All of these methods are effective at reducing network representations to facilitate powerful analysis approaches designed for more compact networks, despite the inherent risk of eliminating important variables in the reduction process. An improvement to these methods, however, would allow analysis on larger networks, and thus reduce the risk of deleting key variables by eliminating the need to oversimplify the networks. In this paper, we propose such an approach.

Wuensche [3] and others also have studied the basins of attraction in Boolean network models of genomic regulation, specifically the relationship of their structures to the stability of attractors (cell types) in the face of perturbations. However, because of the size complexity of basins of attraction, they are often neglected in analysis in favor of the attractor states. As a basin of attraction is a collection of states leading into a corresponding attractor, i.e., phenotype, careful analysis of these basins could reveal interesting biological characteristics that determine cell fate. This is precisely the avenue we pursue in this work.

Willadsen and Wiles [23] form a compact representation of Boolean network state space by creating what they call *schemas*. Using a ternary representation with ones, zeros, and wildcards similar to the don’t-cares of logic minimization, they are able to create an abstract representation of Boolean network basins of attraction, which they use to quantify dynamics and robustness. These schemas provide the authors with a convenient way of representing groups of related, neighboring states as they compute a state space robustness metric called *structural coherency*. While powerful in exploring relationships between state space structure and robustness in random Boolean networks of up to a couple dozen variables, the approach is not intended to identify standout variables that can function as intervention targets.

### Boolean network framework

A Boolean network **B**(*V*,**f**) is made of a set of binary nodes *V*={*x*_1_,*x*_2_,⋯,*x*_*n*_} and a set of functions **f**={*f*_1_,*f*_2_,⋯,*f*_*n*_}. The binary value of variable *x*_*i*_∈*V* at time (*t*+1) is determined by other variables $x_{j_{1}(i)},x_{j_{2}(i)},\ldots ,x_{j_{k_{i}}(i)}$ at time (*t*) by means of a Boolean function *f*_*i*_∈**f**. That is, there are *k*_*i*_ variables assigned to *x*_*i*_, and the mapping *j*_*k*_ :{1,⋯,*n*}→{1,⋯,*n*},*k*=1,⋯,*k*_*i*_ determines the ‘wiring’ of variable *x*_*i*_. Thus, *k*_*i*_ is called the *connectivity* of *x*_*i*_, which is to say the number of inputs to its particular Boolean function. The values of the variables at time (*t*+1) are always a function of the values of the *k*_*i*_ respective input variables at time *t*. Formally,

1 $$x_{i}(t+1)=f_{i}(x_{j_{1}(i)}(t),x_{j_{2}(i)}(t),\ldots ,x_{j_{k_{i}}(i)}(t))$$

#### State transition diagram

**Figure 1:**
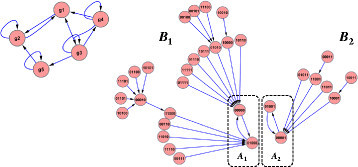
**Five-variable example Boolean network and state transitions.** On the left is the network topology and Boolean function inputs for each variable. Boolean functions for each variable are shown in Table 1. On the right are the 32 states comprising the state transition diagram partitioned into two attractor basins.

The state of a Boolean network at time *t* refers to the state vector for all variables, **x**(*t*)=(*x*_1_(*t*),*x*_2_(*t*),…,*x*_*n*_(*t*)), where a specific state can be expressed as an *n*-dimensional binary vector. The state space of the network is {0,1}^*n*^={00⋯0,00⋯1,…,11⋯1}, whose size is 2^*n*^. Letting **x**(*t*) take on the value of each of the possible 2^*n*^ states and obtaining the next states **x**(*t*+1) gives a set of 2^*n*^ one-step transitions that completely characterize the dynamics of the system. Let this set of all states be called *S*, such that $S=\{x_{1},x_{2},\cdots \;,x_{2^{n}}\}$, and let the set of all transitions between the states of *S* be called *E*. The state transition diagram **G**(*S*,*E*) for a Boolean network **B**(*V*,**f**) with *n* nodes is a directed graph where |S|=|E|=2^*n*^. Each of the vertices **x**∈*S* represents one possible configuration of *x*_1_,*x*_2_,…,*x*_*n*_ and each of the directed edges represents one of the one-step transitions between two states as we synchronously apply Boolean functions to all variables. We choose the synchronous approach [9],[24] over the asynchronous option [25],[26] for its determinism and its origins in relating attractors to biological cell types^a^. The state transition diagram is also called the *basin of attraction field* and more simply as the *state space* of a network. An illustration of Boolean network topology and the state space generated by its functions can be seen in Figure 1 and Table 1.

**Table 1:** **Boolean functions for 5-variable example network**

$x_{j_{1}},x_{j_{2}},\ldots ,x_{j_{k_{i}}}$	***f***_***1***_	***f***_***2***_	***f***_***3***_	***f***_***4***_	***f***_***5***_
00/000	0	1	0	0	0
01/001	1	1	0	0	1
10/010	0	0	0	1	0
11/011	0	0	0	0	0
100	0	0	-	-	-
101	0	0	-	-	-
110	0	1	-	-	-
111	0	0	-	-	-
*j*_1_	2	1	3	3	3
*j*_2_	3	2	4	4	5
*j*_3_	4	5	-	-	-

In the table are Boolean functions for each of the five variables in Figure 1. Input variables are shown at the bottom and output values are shown for each binary combination of inputs.

#### Attractors and basins

In the absence of interventions or perturbations, beginning in any initial state, repeated application of transition functions will bring the network to a finite set of states and cycle among them forever in fixed sequence. This set of states is known as an *attractor*, denoted *A*_*i*_. The complete set of states from which a network will eventually reach *A*_*i*_ is known as the *basin of attraction* for *A*_*i*_, denoted *B*_*i*_. Formally, the states of basin *B*_*i*_ are precisely those that, given *w*≤2^*n*^ applications of Boolean functions to an evolving state, end up in attractor *A*_*i*_: *B*_*i*_={**x**∣*f*^(*w*)^(**x**)∈*A*_*i*_},*i*,*w*≤2^*n*^. The basins of attraction correspond precisely to the *weakly connected components* of the state transition diagram (i.e., a directed subgraph such that every pair of vertices *u* and *v* is connected either by a directed path from *u* to *v* or a directed path from *v* to *u*), and the attractors correspond precisely to the *strongly connected components* of the state transition diagram (i.e., a subgraph such that every pair of vertices *u* and *v* is connected by a directed path both from *u* to *v* and also from *v* to *u*).

An individual basin *B*_*i*_ and its attractor *A*_*i*_ can be described in terms of the collection of states comprising them, $A_{i}\subseteq B_{i}\subseteq S=\{x_{1},x_{2},\cdots \;,x_{2^{n}}\}$. Let the size of each attractor ∥*A*_*i*_∥=*p*_*i*_, where *p*_*i*_ is the period, or length of the attractor cycle. An attractor with *p*=1 is called a point, or singleton attractor, and an attractor with *p*>1 is called a cyclic attractor (with cycle length equal to *p*). If **x**_*i*_ is a state in *A*_*i*_, we can describe the next state of a point attractor as **x**_*i*_(*t*+1)=**x**_*i*_(*t*), and the behavior of a cyclic attractor as **x**_*i*_(*t*+*p*)=**x**_*i*_(*t*). Boolean networks may have anywhere from one cyclic attractor comprised of 2^*n*^ states to 2^*n*^ point attractors, although most commonly a network will have just a handful of singleton or short-cycle attractors.

All attractors are subsets of their basins (i.e., *A*_*i*_⊆*B*_*i*_,∀_*i*_), all basins (and concordantly all attractors also) are mutually exclusive (i.e. $B_{i}⋂B_{j}=\emptyset ,\forall_{i\neq j}$), and the complete state space is comprised entirely of all basins (i.e., ${⋃}_{i}B_{i}=S$). For referencing specific basins and attractors, the set of all basins is denoted **B**={*B*_1_,*B*_2_,⋯,*B*_*L*_}, and the set of all corresponding attractors is denoted **A**={*A*_1_,*A*_2_,⋯,*A*_*L*_}⊆*S*,1≤*L*≤2^*n*^.

### Previous work

Here, we briefly describe pertinent points of our previous work [5] upon which the current methods and results build.

#### Logic minimization

Logic minimization (or reduction) is a classic problem from digital circuit design employed to reduce the number of actual logic gates needed to implement a given function [27]. With careful logic minimization, one can reduce the number of gates required and thus include more functionality on a single chip. Minimization identifies variables which have no influence on the outcome of a function and marks them appropriately as a *don’t-care*. As a simple example, we take the Boolean function: (*A*∧*B*)∨(¬*A*∧*B*) (two signals, four gates). Since the role of *A* changes while *B* remains *ON* with the same output, it is clear to see that the only influencing variable is *B*, which can be given with just that signal itself (0 gates).

We employ the Espresso tool [28], which is a heuristic logic minimizer designed to efficiently reduce logic complexity even for large problems. We supply as input the set of states in a particular basin of attraction *B*_*i*_ (the complete state space is comprised entirely of all basins (i.e., ${⋃}_{i}B_{i}=S$)); this input comprises the *ON-cover* (or truth table) in disjunctive normal form (DNF) for a Boolean function whose output is *ON* for the states of *B*_*i*_ ($\{x_{i_{1}}\vee x_{i_{2}}$$\vee \cdots \vee x_{i_{M}}\}\mapsto \text{ON}$) and whose output is *OFF* for the states of *S*∖*B*_*i*_. Espresso analyzes this cover and returns a minimal (though not necessarily unique) DNF set comprised of one or more terms, denoted $T_{i}=\{t_{i_{1}},t_{i_{2}},\cdots \;,t_{i_{N}}\}$, where *N*≤*M*. These **t**_*i*_ have some variables set to *ON* (denoted 1), some set to *OFF* (denoted 0), and some set as *don’t-care* (denoted ‘-’). The presence of these don’t-care variables in some terms is what allows the reduction.

For a reasonable number of variables, enumerating all 2^*n*^ states in the state transition diagram is not an issue. By starting at each state and evolving the network forward, each attractor and its basin can be enumerated. Exhaustive enumeration is the best possible situation for logic minimization because with more states, more common values can be identified and summarized in the reduction. In contrast, a partial enumeration obtained by a sampling approach greatly hinders the reduction step and results in many remaining terms with fewer don’t-cares. Enumerating the full state transition diagram runs in time exponential in *n*, specifically *O*(2^*n*^), due to computing the next state for each of the 2^*n*^ states.

#### Single-variable intervention targets

We next review measures first introduced in our initial work [5] for finding single variable intervention targets. The first measure describes how frequently a variable *v* is required to be *ON* or *OFF* across different terms, called *Popularity* (*p*(*v*)=*x*/*y*), where *x* represents the number of times *v* is set in a term *T*_*i*_ and *y* represents the total number of terms in *T*_*i*_. Next, we identify terms which are powerful due to the combinatorial effect of their few set variables over the remaining unset variables. *Term power* is defined as (*P*_*T*_(**t**)=1−*a*/*n*), where *a* is the number of variables set in a term (**t**) and *n* is the number of variables in the network. One can also consider variables which preside over powerful terms to make excellent candidates for intervention targets. *Variable power* is defined as the average term power over the terms in which a variable *v* is explicitly configured, where *b* is the number of terms where *v* is set and *y* is the total number of terms in *T*_*i*_, namely, $P_{V}(v)=[\;\overset{y}{\underset{i=1}{\sum }}P_{T}(t_{i})|(v\;\text{is}\;\text{set}\;\text{in}\;t_{i})]/b$.

We have found that for networks of a size manageable enough to exhaustively enumerate the state space, popularity and variable power can be used to identify key variables which make excellent candidate intervention targets. As described in the Additional files, we have performed a simulation experiment to identify the best single-variable measures between popularity, power, and related measures described in our previous work [5] [See Additional file 1]. We found in a simulation study over thousands of random networks with between 7 and 16 variables, that popularity, power, and their combination in the form of an harmonic mean^b^ showed the most statistically significant differences in intervention success rates of all 14 methods compared. Included in the comparison were Boolean network measures as well as graph theoretic measures.

Unfortunately, however, larger networks present some problems for these measures and require a different approach. The problems in larger networks manifest because popularity and power depend heavily upon the reducibility of the basin of attraction field by logic minimization. In larger networks, where we are forced to explore the basin of attraction field by sampling, the reducibility of the state space is greatly hindered and our single-variable measures are rendered unusable.

In this work, we contribute a multiple variable intervention target called a template and show how, even in large networks with sampled state spaces, that they are still able to identify powerful targets for intervention. Thus, we see the template approach effectively taking over for the former single-variable measures, especially in larger networks. Finally, we contribute an example templates application to a T-LGL leukemia network and analyze the implications of our approach on this real world scenario.

## Methods

As the number of variables in a biological interaction network increases, the more likely it is that a successful intervention will require multiple variables. In fact, in our work in AI planning [18], we found that a planned sequence of interventions was an effective way to transition to a desired steady state. Our previous measures of popularity and power are capable of identifying multiple high-value intervention targets separately in smaller networks. In this section, we will introduce intervention templates to take into account the multivariate effects of gene regulation and propose an approach to address larger networks. We will be faced with several challenges such as: (1) how to consider many subsets of variables in each basin of attraction, (2) to design clear methods and measures to identify the best template-based intervention targets in a systematic way, and (3) to work with an intractable state space through sampling to cope with larger networks. We end by outlining a robust simulation study designed to illustrate achievement in these three areas.

### Template-based intervention targets

Let the term *template* indicate a subset of variables (ordering not important) in a specific 0/1 (*O* *F* *F*/*O* *N*) configuration. Let the term *k-template* refer to a template with *k* variables; call the maximum value of *k* being considered *K*. Thus, for *n* variables there exist ∑k=1K2knk templates. This follows from nk ways of selecting *k* unique sets of variables from *n* total, 2^*k*^ binary value combinations for each of those sets, and *K* values of *k*.

Since a template is a subset of *n* network variables assigned to a specific Boolean configuration, each template with *k* variables covers 2^*n*−*k*^ other states. The smallest extreme is a template with *k*=1, or a 1-template, which is a single-variable assigned to *ON* or *OFF*. The largest extreme would be a template with *k*=*n* — i.e., a single state in the state space. Such a template would cover no additional states, would not provide any further insights, and would be trivial to count. In practice, *k* is typically small, in the range 1 to 5 depending on *n*. Because there are (2k)nk templates for every *k*, counting (and studying) quickly becomes intractable. However, this is not typically an issue when seeking to identify intervention targets in biological networks since the difficulty of intervening increases with the number of variables required in the actual intervention.

Our combinatorial analysis involves counting the occurrence of each template remaining in the minimized DNF terms (*T*_*i*_) of the original basins of attraction, (*B*_*i*_), and is described in Algorithm 1. Due to properties of the binomial coefficient [29], the algorithm executes with a runtime exponential on *n* and the size of the templates^c^.





#### Template-based scores for intervention target selection

With the vast number of templates, we require ways to identify the important, top templates most likely to make the best intervention targets. After counting the occurrence of all templates in all basins, we begin by analyzing the most frequently counted templates as potential top intervention targets. Since logic minimization can greatly reduce the representation of the attractor basins (and thus the overall template counts), we provide a second measure to identify top templates with frequencies diminished by the logic minimization step.

While we apply our measures to templates of all considered sizes, there is not an explicit penalty applied to larger templates. This decision is motivated by the fact that all interventions must be interpreted and evaluated in context, since it may be that a ‘larger’ intervention could involve easier-to-target genes and/or be less invasive than a smaller intervention, or that a seemingly ideal smaller intervention may not be biologically or medically possible. Thus, we report the best templates over several smaller sizes and leave the translation from mathematically best targets to medically best targets to domain experts who, we hope, would prefer the least invasive options.

*Template frequency (* *F* *)*: the most frequently counted templates in a particular basin are the first place to look for templates likely to make top intervention targets. By examining the set of terms *T*_*i*_ (reduced states with some don’t-care variables) of a basin *B*_*i*_, we can, for each of the j=1:(2k)nk templates, count how many times that template appears in the terms of *T*_*i*_. Formally, *F*_*j*,*i*_ is the final count of occurrences of template_*j*,*i*_ in the set of terms *T*_*i*_ (corresponding to basin *B*_*i*_). The maximum value of *F*_*j*,*i*_ is 2^*n*−*k*^ (i.e., the total number of Boolean states covered when the *k* variables of the template are fixed and none of the remaining *n*−*k* variables have been eliminated by logic minimization), though in practice, the value of *F*_*j*,*i*_ is much lower, especially when state spaces are exhaustively enumerated and then greatly reduced by logic minimization. We can rank templates by *F*_*j*,*i*_ to find top template candidates.

*Template basin distribution percentage (* *D* *)*: just because a *k*-template is the most frequently counted template in a particular basin does not necessarily mean that it is the most significant. Sometimes, especially when logic minimization is able to significantly reduce the number of states in a basin to a much smaller set of terms, the frequencies of important templates will be diminished due to the introduction of many don’t-care values. Thus, we need a way to identify these high-value templates despite their lower frequencies of occurrence. To do this, we consider the distribution of a particular template across all basins, or in other words, the affinity of a template to a particular basin. Since templates can and often do appear in terms of multiple basins, we will calculate the ratio of occurrence in each basin and of the total number of occurrences. Formally,

2 $$D_{j,i}=\frac{F_{j,i}}{\sum_{m=1}^{L}F_{j,m}},$$

where *L* is the total number of basins, *j* is the template number, and *i* is the basin number. Like frequency, for each basin, we can rank the templates by this ratio to find top template candidates.

These template measures provide two ways to identify a subset of templates warranting further investigation. By analyzing templates among the highest values of *F*_*j*,*i*_, we will identify variables occurring together in a particular basin most often. By analyzing templates among the highest values of *D*_*j*,*i*_, we will identify variables that may not be the most frequent but retain the most affiliation with a particular basin even after logic minimization.

#### Intervention targets

For each basin of attraction, full or partial, we can compute the best intervention targets using the following 11 methods. The first three methods comprise the best of the previous small network measures, namely, popularity, power, and their harmonic mean (abbreviated POP, POW, and HPP). The next four are the top templates of sizes 1, 2, 3, and 4 computed according to *D* (abbreviated K1TBDP, K2TBDP, K3TBDP, K4TBDP). The final four are the top templates of sizes 1, 2, 3, and 4 computed according to *F* (abbreviated K1FREQ, K2FREQ, K3FREQ, K4FREQ). For the simulation study described later in this section, we compare the single best template identified by each of these 11 methods. For our application to T-LGL leukemia, we examine the sets of top templates identified by K1FREQ, K2FREQ, and K3FREQ.

#### Illustration

**Figure 2:**
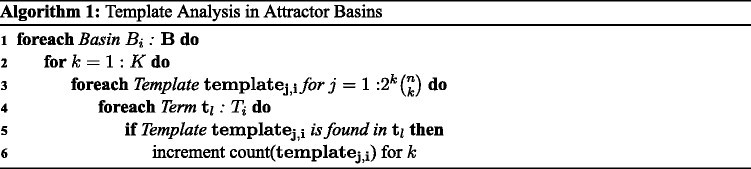
**Example of Boolean network basin state minimizations.** On the left are two boxes containing each state in the two basins shown on the right of Figure 1. On the right are the terms produced from applying logic minimization to each basin. The larger basin is reduced to two terms with one value each (OR relationship between the variables) and the smaller basin is reduced to one term with two variables set (AND relationship between the variables).

Let us consider for now the *unreduced* state space of the five-variable example network shown in Figure 1 and observe how to identify templates and how those with high *F* and *D* values can be used as interventions. The states from the diagram are collected and listed in Figure 2 (left). We will count a few specific templates visually from the complete set (on the left), but later on in practice, we will count templates from the set reduced by logic minimization (on the right). We can quickly see from the dramatic reduction (Figure 2 (right)) that *g*_3_ and *g*_5_ are the key players in the network and that they display contrasting behavior between the two basins. As such, let us examine only 1- and 2-templates involving *g*_3_ and *g*_5_ and observe their frequency and template basin distribution patterns. We begin with *k*=1, and follow its discussion with *k*=2.

We will first consider the four 1-templates involving *g*_3_ and *g*_5_ and look for any disproportionate patterns among the two basins. By inspection of the states of *B*_1_, we can quickly see that each of the 16 occurrences of *g*_3_=1 and of *g*_5_=0 are counted there and that these are the only 1-templates with a maximal count of 16 in either basin. These counts are contrasted in *B*_2_ where we find the 1-templates for *g*_3_=0 and *g*_5_=1 (opposite configuration) counted the maximum of eight times (since there are only eight states in the basin). With no logic reduction in this example, these *F* values produce proportional *D* values that elevate *g*_3_=1 and *g*_5_=0 for *B*_1_ and *g*_3_=0 and *g*_5_=1 for *B*_2_.

There are 40 countable 2-templates (2^2^ combinations of 52=10 templates) with a maximum frequency of 8 for each template. We next consider just the 2-templates involving *g*_3_ and *g*_5_. *B*_2_ counts just one of the 40 total 2-templates the maximum of eight times - precisely the template remaining after logic reduction: *g*_3_=0 and *g*_5_=1. *B*_1_ counts 15 of the 40 total 2-templates eight times, 12 of them six times, 12 of them four times, and 1 of them zero times, but counts none exclusively. Of the 15 templates counted the maximum of eight times in *B*_1_, all templates for *g*_3_ and *g*_5_ complementary to those counted in *B*_2_ are among them.

With the increased value of *k*, we observe more dramatic template basin distributions and now reveal a template with complete affinity for *B*_2_, which we did not see for *k* = 1. We now find all eight 2-template occurrences of *g*_3_=1 and *g*_5_=0 simultaneously and exclusively in *B*_2_ (100%), and this is corroborated by the logic minimization of *B*_2_ which left us one single term with the same variables remaining in the same values we now find. Thus, we observe that the templates with the highest *F* and/or *D* values correspond to the variables shown to exhibit the most network influence by logic minimization. Concordantly, while a single-variable intervention is able to transition to *A*_1_ from any starting state in the network, we observe that a transition to *A*_2_ from anywhere in the network requires the multi-variable intervention revealed by our highest frequency template for *B*_2_. Since single-variable measures based on the results of logic minimization produced good targets in our previous work, we are motivated to further investigate the intervention viability of templates given their correlation with logic minimization results.

### Sampling large state spaces

As the size of the network grows, exhaustive enumeration of the state space (size 2^*n*^) quickly becomes intractable and a sampling approach is required. While Wuenche’s method of directly computing pre-images [30] allows exhaustive state space enumeration for up to 31 variables with the DDLab software [31] (and even >31 for single attractor basins), our implementation of state space enumeration begins to suffer performance degradations after 22 variables. Because we are interested in networks well beyond 31 variables, we transition to a sampling strategy whereby we randomly and uniformly sample a number of initial starting states from the state space range [ 1:2^*n*^]. From each starting state, the network is then run forward to the corresponding attractor, collecting any states visited along the *transient* path. Attractors are noted and all corresponding states are collected into a partial basin, and it is from these partial basins that we identify our intervention targets. This approach will sometimes miss attractors with very small basins leading to them, but it certainly finds the largest ones, and for a large number of samples, gives us a significant set of member states to analyze. We can also approximate the percentage of the total state space occupied by each basin based on the percentage of total samples associated with it. It should be carefully noted that when sampling, the identified partial basins are themselves proper subsets of the complete basins. In other words, the sampling approach creates no incorrect assignments of basin states to attractors. With exhaustive enumeration, we complete the state space exploration and acquire all states in each basin. Thus, both sampling and exhaustive enumeration provide correct basin states, just with sampling being an incomplete picture and exhaustive being a complete picture.

### Evaluating intervention success

Abstractly, an intervention should shift the steady behavior of a system to a different (usually more desirable) state. In the context of the Boolean network formalism, this is represented by shifting the steady behavior of the system, represented by an attractor state or cycle, into a different basin of attraction. Specifically, our intervention goal is to identify minimally sized templates that reliably transition the network from undesirable attractors to desirable attractors. Depending on the patient and the biology of the identified template variables, this could mean preferring smaller templates (possibly less invasive) with a lower chance of success or choosing slightly larger templates (possibly more complex) with a higher chance of success. A successful intervention needs only to shift the state from a starting attractor state into any state in the basin of the goal attractor, as the network dynamics will then naturally bring the state to the attractor itself.

With the top intervention template candidates determined by *F* and *D* for each basin, we estimate intervention success rates by attempting interventions to each basin as a goal destination, starting from attractor states from each outside basin, recording each attempt as a success or failure. Across many Boolean networks, we will find a range of the number of attractor basins, from one or two to dozens, and within those basins, attractor cycles of various periods, from 1 to 5 to 100 or more. In estimating intervention success, we should not let abnormally large attractor cycles bias the results by providing too many of the intervention starting states. For example, if we have a network with a point attractor in one basin and an attractor cycle of 50 states in the second basin, our intervention success estimates would be based on an unfair distribution of starting states. In practice, if an attractor cycle is longer than ten states, we randomly sample ten attractor states for that basin upon which to apply candidate interventions. If it is less than ten, we use them all.

In our simulation study (described next), we must compare the performance of top intervention methods across various network sizes and between the methods themselves. As we will be comparing pairs of success proportions for independent interventions, which qualify as Bernoulli trials under the binomial model, we will use a two-proportion *Z*-test with the null hypothesis of having equal success proportions [32].

### Simulation study

Our two main challenges with the template approach involve performance as we transition from fully enumerated state spaces to sampled state spaces and also performance as we increase the size of the networks. To address these concerns, we design a simulation study over hundreds of randomly generated Boolean networks within which we compare performance of the former single-variable measures and the new template-based approach.

#### In-silico network models

In order to test interventions over an adequate range of network sizes, we create multiple random networks with 10, 12, 18, 20, 25, and 40 variables each, for a total of 200 networks. In each network, we randomly generate Boolean update rules, which creates random network connectivity as we randomly choose *k*_*i*_ inputs for each variable *x*_*i*_. In order to create biologically inspired networks, we adopt the per-variable connectivity distribution from Albert and Othmer [6] used originally for a *D. melanogaster* network, shown in Table 2. Once the inputs are chosen for each variable, random Boolean functions are formed by generating random and independent zeros or ones for each binary input combination. Thus, all $2^{2^{k_{i}}}$ Boolean functions are possible in our random networks.

**Table 2:** **Connectivity distribution for random networks**

Number of inputs	Probability
1	0.101
2	0.233
3	0.267
4	0.183
5	0.083
6	0.083
7	0.050

In the table, we see the probability of assigning various numbers of inputs to random Boolean functions.

Once network connectivity and rules are determined, the basin of attraction fields must be generated in full or in part. For the networks of size 10, 12, and 18, we use half the networks for exhaustive enumeration of the basins of attraction, and for the other half of networks, we enumerated partial basins through sampling. For networks of size 20, 25, and 40, only sampled state spaces were used.

#### Performing interventions

An intervention is defined as a modification (set/reset) to one or more variables in an attractor state of a source basin with the intention that network rules will transition to any state in a given goal basin (thus eventually reaching the attractor of the goal basin). For the simulation study, we do not attempt interventions where the goal and source basins are the same, since these are more likely to succeed and would inflate our results. Likewise, we do not attempt interventions to goal basins estimated to occupy less than 15% of the total state space since reaching these very rare basins is the most difficult and has little biological relevance. Our 200 original random networks, through their various numbers of attractor basins and attractor cycle lengths, produced 4,223 individual intervention attempts, each applied separately with all 11 methods.

## Results

Next, we present the results of the simulation study described in the Methods section, which reveals the ability of template-based interventions to maintain performance between exhaustive and sampled state spaces and also in increasingly large random networks with sampled state spaces. We then provide a demonstration of the approach on a real-world network modeling T-LGL leukemia, originally hand-created by domain experts.

### Simulation study for template methods

In order to demonstrate the robustness of template-based interventions, we present the results of the simulation study described in the ‘Methods’ section. The study addresses two main questions: (1) what effect does the change to sampled Boolean network state spaces have on the performance of template-based interventions? and (2) what effect does increasing network size have on the performance of template-based interventions? To address these questions, we analyze hundreds of randomly generated Boolean networks for which we compare performance of single-variable measures and the variations of the new template-based approach.

#### The effect of sampling on template interventions

**Figure 3:**
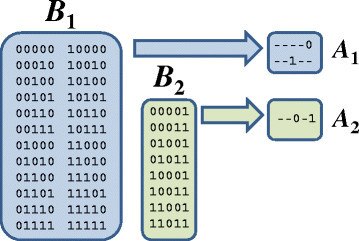
**Success rates between exhaustive and sampled state spaces.** For three network sizes, we see proportions of successful interventions across single-variable and template-based approaches. The first three sets of bars in each subfigure are the single-variable measures of popularity, power, and their harmonic mean. The latter eight are the two template approaches of *D* and *F* across four template sizes. *P* values reflecting proportion differences can be found in Table 3.

To address the challenge of whether or not template-based approaches remain robust as we transition from a fully enumerated state space to a sampled one, we compare the performance of each measure between full and sampled versions of the 10, 12, and 18 variable networks. Since these networks are small enough to exhaustively enumerate the full state space, the comparison will provide a full assessment of any performance degradation due to sampling. For each network size, we compare the proportions of successful interventions of each type between exhaustive and sampled networks. Interventions that show a significant change in proportion between exhaustive and sampled state spaces will be noted. Success rates can be seen in Figure 3 (with Figure 3 significance values in Table 3), for which we note the following observations:

**Table 3:** ***P*** **values for two-proportion** ***Z*** **-tests**

	10E/10S	12E/12S	18E/18S
POP	0.0920	0.4619	0.0000
POW	0.0013	0.0003	0.0000
HPP	0.0000	0.2030	0.0000
K1TBDP	0.0000	0.0000	0.0000
K2TDBP	0.0007	0.4421	0.0000
K3TDBP	0.0002	0.4391	0.0000
K4TBDP	0.5424	0.1385	0.0000
K1FREQ	0.2197	0.0002	0.2038
K2FREQ	0.0004	0.0000	0.3964
K3FREQ	0.2015	0.0000	0.0192
K4FREQ	0.0062	0.0014	0.2310

In the table, we see the *P* values for two-proportion *Z*-tests across 11 intervention targets between exhaustively enumerated (E) and sampled (S) networks with 10, 12, and 18 variables. For *P* values below the ***α***=0.05 significance level, we reject the null hypothesis and conclude that there exists a statistically significant difference in success proportions between the exhaustive and the sampled state space cases. These differences can be inspected visually in Figure 3.

In the networks with 10 variables, 9 of 11 methods showed a decrease in performance from exhaustive to sampled, with 6 of those being statistically significant changes. Interestingly, 2 of the 11 methods showed an increase in performance, with one of those being statistically significant at the 0.05 alpha level; both of those cases involved frequency-based templates. While showing decreases in performance between exhaustive and sampling with each template size, template basin distribution percentage templates registered among the highest success proportions overall, indicating that they might be a suitable replacement for single-variable methods in exhaustive cases if the computational cost of templates can be afforded.

In the networks with 12 variables, all four template sizes for frequency-based templates showed an increase in performance in the sampled cases, with all four differences in proportions being statistically significant. All seven other methods showed visible decreases in performance, with two of them being considered statistically significant. Again, template basin distribution percentage templates had among the best performances in the exhaustive cases.

In the networks with 18 variables, we see the clear resistance of frequency-based template approaches to the side effects of sampled state spaces. With one statistically significant increase in performance and no significant decreases, frequency-based templates overwhelmingly dominate performance in sampled cases while all other methods drastically decrease.

It is very interesting to observe any increase in performance between a fully enumerated space and a sampled space. In our case, we observe this behavior because, in the sampled condition, the intervention targets are computed from a larger amount of terms. This is due to the fact that the logic minimization ability is highest when every state is known beforehand and it is hindered greatly by not knowing all basin states ahead of time. Only the templates (especially the larger ones) benefit from this situation - single-variable measures like popularity and power suffer when there are *fewer* don’t-cares in the terms, and some template measures suffer when there are *too many* don’t-cares (i.e., exhaustive case). Thus, while drastic logic minimization on exhaustive state spaces allows popularity and power to quickly reveal decent targets, a less effective logic minimization leaving many more terms behind after reduction benefits the template approach by providing more information from which to identify the best templates while still eliminating the least important variables. In some cases, this benefit outweighs the benefit of a full logic minimization.

While we expected to avoid recording, for template approaches, significant success proportion decreases for sampled network state spaces, we not only failed to detect that trend altogether in frequency-based templates but also in many cases detected significant increases. We also began to see the template basin distribution percentage templates as most ideally suited to smaller networks in exhaustive cases, perhaps as a more thorough alternative to the former single-variable measures. From these data, we conclude that frequency-based template methods are much more robust in sampled state spaces than their single-variable counterparts. The next step is to observe if this trend continues with increasing network size.

#### The effect of increasing network size on template interventions

**Figure 4:**
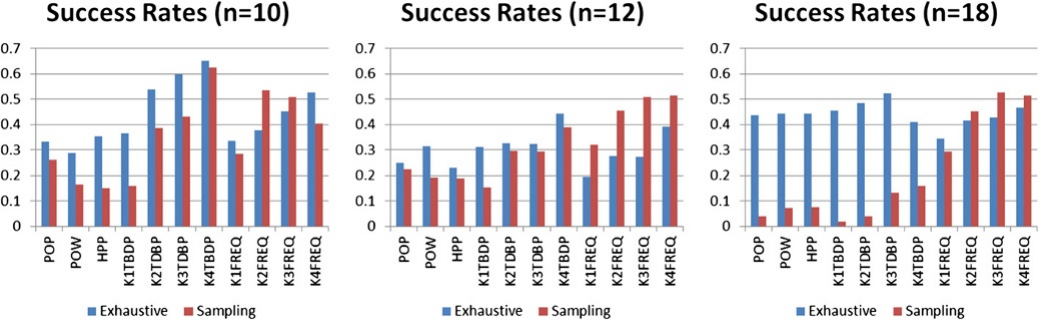
**Success rates for** ***D*** **and** ***F*** **templates.** **(A)** We see the proportions of successful intervention attempts for *D*-based templates of four sizes across six different network sizes. **(B)** We see the same for *F*-based templates. Both subfigures include the performance of the single-variable approaches for comparison. While *D*-based templates perform reasonably well in the smallest network sizes, it is clear that only the *F*-based templates show consistent and significant performance across all network sizes. Note that 4-variable templates are not included on the 40-variable network for computational reasons.

Satisfied that template measures remain robust in networks with sampled state spaces, we now investigate larger random networks. It is expected that any measure or technique will decrease in performance as the size and/or complexity of the network increases. However, with the knowledge that the single-variable measures fail completely with sampled state spaces even for small networks, we need to be assured that template performance remains robust. We generate further random networks with 20, 25, and 40 variables, which produce state space sizes of 2^20^, 2^25^, and 2^40^. We sample these enormous state spaces with about 1% or less coverage in initial states and estimate the intervention success of the top scoring intervention targets from our 11 methods (3 classic single variable, 4 sizes of *D* templates, and 4 sizes of *F* templates). For the 40-variable network, we do not include size-4 templates for computational considerations of the simulation. In Figure 4A, we show the performance of template basin distribution (*D*)-based templates at template sizes of 1 to 4 and the single-variable measures of variable popularity (POP), variable power (POW), and the harmonic mean of the two measures (HPP). In Figure 4B, we show the performance of frequency (*F*)-based templates of sizes 1 to 4 against the same single-variable methods. Both subfigures show performance over networks of size 10, 12, 18, 20, 25, and 40, all with sampled basins of attraction. Error bars shown reflect the 95% binomial confidence intervals.

In Figure 4A, we observe that template basin distribution percentage templates do not consistently show significant differences in success proportions with POP, POW, or HPP. We do note that K3TDBP and K4TBDP do show significant performance over POP, POW, and/or HPP in all networks up to 25 variables, but they are significantly outperformed by their frequency-based counterparts in Figure 4B. In Figure 4B, we observe that the only time a frequency-based template does not show a significant difference in proportion is in the 10-variable network for the template with only one variable (the most extreme case); though in practice, we would not apply template analysis on such small networks. A complete separation of 95% confidence intervals surely indicates a significant separation in success proportions [33], but to further reinforce these observations and to reveal any significant differences in proportions not obvious from confidence intervals, we computed two proportion *Z*-tests for independence for each pair of methods for each network size. These pairwise matrices of *P* values revealed even stronger conclusions than the graphs in Figure 4A,B, further confirming the statistically significant differences beyond what is obvious by visual inspection of the error bars. As they do not reveal any critical trends not visible in Figure 4A or B, we reserve these *P* value matrices for the Additional files [See Additional file 2].

#### Summary of simulation study

Over all network sizes between *F*-based and *D*-based templates, it is clear that *F*-based (frequency) templates not only maintain performance between exhaustive and sampled networks but also provide consistent success rates with increasing network sizes despite the exponential explosion of state space sizes. We were also interested to observe that in some cases, the inhibited reducibility of sampled state spaces actually contributed additional information to the computation of the larger template targets - in some cases actually improving their performance in sampled networks over their performance in maximally reduced state spaces. Template basin distribution percentage-based templates are sometimes useful in smaller networks and are the most effective in smaller, exhaustive networks as a more thorough alternative to the simpler, single-variable measures of POP, POW, and HPP, albeit at an increased computational cost.

### Application to T-LGL leukemia network

In our previous work [5], we identified useful intervention targets using the single-variable measures in real-world networks for melanoma, the yeast cell cycle, and for human aging. Because we saw in our simulation study that no significant new information is revealed by template approaches in exhaustive state spaces, we do not apply our template approaches to those previously explored networks here. Instead, because of the robustness of the templates approach for large networks with sampled state spaces, we make application to a 43-variable network for large granular lymphocytic (T-LGL) leukemia where the single-variable measures have no usefulness.

Zhang et al. [34] have methodically constructed a model of the blood cancer T cell large granular lymphocyte (T-LGL) leukemia from hundreds of literature sources. The original study, as well as others based on variations of this large network [21], have searched for therapeutic targets and have even validated some experimentally. However, these predictions required expert-level topological reduction and simplification of the network in various ways. But because of the validated findings, this network makes an ideal situation in which to apply our approach, which is purely computational and requires no expert-level knowledge of the disease system. If our results on the larger, less simplified network are reasonable, our approach will be shown useful and applicable on large networks for which we may not have expert-level knowledge and/or the ability to systematically simplify.

#### Network construction

**Figure 5:**
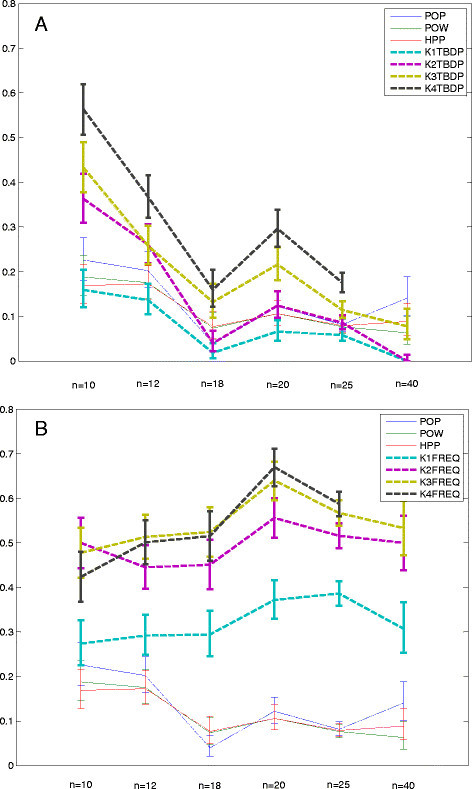
**43-variable T-LGL leukemia network.** 43-variable T-LGL leukemia network after simplifications described in the Additional files [See Additional file 3].

The original network [34] created from the literature contained 128 nodes and 287 edges but was simplified by the authors through software and manual adjustments to 60 nodes and 142 regulatory edges. After collaborating with a principle author from [34], we performed further reductions on the network according to techniques described in related work involving this same network [21],[35]. The goal of further reduction was to remove nodes which mask the dynamic behavior of the network variables (i.e., the overarching influence of the apoptosis node as well as control nodes); since steady-state analysis will be performed over many randomly generated states, control variables are not necessary since variables they control will be forced to take on different values through random starting state assignment. After the simplifications described in the Additional files, we obtain the 43-variable version shown in Figure 5 [See Additional file 3]. A list of the Boolean functions is also given in the supplements [See Additional file 3].

#### Network and state space properties

With 50,000 randomly and uniformly sampled initial states, the partial basin of attraction field was enumerated, resulting in five basins of attraction, four of which comprise greater than 99% of the state space (estimated), and will thus be the focus of the analysis. These four basins are summarized in Table 4. In other sampling, runs up to seven attractors were identified, but these additional two, when discovered, were estimated to occupy thousandths, if not tens of thousandths of 1% percent of the state space and would be discarded for analysis along with the fifth basin. Due to the massive size of the full state space (2^43^, over 8 trillion states), 50,000 initial sampled states was chosen first due to it being large enough to proportionally reveal all major basins and, second, because choosing more samples, such as 100,000 or 200,000, would only marginally increase the coverage of the full state space.

**Table 4:** **Summary of four attractor basins for the 43-variable leukemia network**

	Basin	Attractor	Number of	Estimated %	Attractor
	number	period (***P***)	states	coverage	type
	1	4	58713	40.88	Healthy
50,000 Sampled + 93,618	2	1	57359	39.94	T-LGL
Transient = 143,618 Total states	3	1	17099	11.91	T-LGL
	4	4	10441	7.27	Healthy

We observe the attractor length, numbers of states identified, and the estimated state space coverage for each basin. Attractor type classification criteria are described in the text.

We have categorized the four basins to either healthy (i.e., normal apoptosis function) or T-LGL (i.e., cancer state) based on the values of certain key variables in the steady attractor states/cycles. In the original 60-variable network, the presence of control nodes, including one for apoptosis, simplified classification of attractor states. Since the apoptosis node was wired to nearly every other node for purposes of the original study [34], its behavior dominated the dynamics of the entire network. Since we have stripped the network of the apoptosis node, as well as other control nodes, we must interpret the attractor states based on other criteria. These criteria involve precisely the series of input regulatory nodes controlling the former apoptosis node. Nodes found in identical steady states across all four attractors were not considered, but several variables, namely FasT, Fas, Ceramide, and FLIP, were used in their boolean functions to effectively determine whether or not an apoptosis node would have been active or inactive in the attractor. Full attractor states are given in Additional file 3. Here, we have four attractor basins, with two classified as healthy and two as T-LGL. Among the two in each category, one exhibited stronger, more consistent behavior. Specifically, Basin 1, as the largest basin in the space, was classified as healthy but had oscillatory values for DISC and FLIP, direct influences on apoptosis. Basin 4, on the other hand, had consistent behavior for both DISC and FLIP despite the cyclic nature of the attractor. Thus, due to the small size of Basin 4 and its pure behavior even with a cyclic attractor, it is considered the healthiest attractor. For complimentary reasons, Basin 2 is the larger and thus more general T-LGL attractor, and Basin 3 is the smaller, more precise T-LGL attractor. A total of 11 attractor states were identified: a cycle of 4 states in Basins 1 and 4, and singleton attractor states in Basins 2, 3, and 5, the latter of which was not considered as a goal state.

#### Templates analysis

Next, we identify key variables from *k*-templates for *k*=1,2, and 3. The 43 network variables give 86 1-templates, 3,612 2-templates, and 98,728 3-templates to count across the minimized terms in our four basins. We stop with *k*=3 since *k*=4 provides nearly 2 million 4-templates to count, but only across less than 120,000 terms. Future work intends to parallelize and split up the computational burden of the sequential counting algorithm and offer conditions under which we can test the efficacy of counting orders of magnitude more templates across relatively few terms.

After counting *k*-templates for *k*=1,2, and 3, we estimated the intervention success rate for the 30 most frequent templates (highest values for *F*_*j*,*i*_) in each size and for each basin. Full listings of these templates can be found in Additional file 3, but a listing of interesting templates is found here in Table 5. To estimate a success rate, we apply each top template intervention to all 11 attractor states across all basins in the T-LGL network and compute the distribution of basins reached. If a template causes a significant number of these attractor states to jump to (or remain in) a desired basin, such a template is of great interest. In general, we expect an attractor state to remain robust to perturbation but expect the larger templates to have the best chance at changing the steady state of the system. Since Basins 1 and 4 each contain a four-cycle of attractor states, we expect at least 4 of the 11 destination states (36%) to remain in those basins (due to the expectation of robustness mentioned). Likewise, the remaining basins each have a single attractor state each and we expect 1 of the 11 destination states (9%) to remain in these basins. Of the top 30 templates for each *k* and each basin, we indeed saw these expected distributions of goal states very frequently. Numbers above and beyond these expectations warrant closer inspection, which we provide for the most interesting templates listed in Table 5.

**Table 5:** **Notable leukemia network templates**

Basin	Rank	Variables	Values	***F***_***j,i***_	Success
1	18	Ceramide	0	22,936	0.73 ^a^
		PDGFR	1		
		S1P	0		
2	27	PDGFR	0	28,057	1.00
		S1P	0		
		TBET	1		
3	1	Ceramide	0	14,404	1.00
		PDGFR	1		
		S1P	1		
4	6	TBET	0	6,404	0.73

^a^Remaining 27% of interventions lead Basin 4 (also healthy). Listed in the table is a selection of interesting templates from the analysis of the leukemia network. Complete lists of the top frequency templates for each basin and each template size can be found in Additional file 3.

While the simulation study was clear that intervening with a template of high frequency was sufficient to best comparable methods, before continuing, we provide a comparison demonstrating that more successful templates and fewer powerless templates are found among the most frequently counted. To do this, we compute success rates for 3-templates in three categories: the 1,000 most frequent, 1,000 random, and the 1,000 least frequent templates. These rates are shown in Table 6 and, as expected, we see with statistical confidence that the top 1,000 most frequent templates have higher proportions of success than the other groups.

**Table 6:** **Frequent templates are more successful than others**

	Top 1,000	Random 1,000	***Z***-score	***P***value
Basin 1	4,493	4,293	2.753	0.00298
Basin 2	3,838	1,783	31.767	0.00000
Basin 3	2,534	1,073	26.605	0.00000
Basin 4	4,520	3,378	16.050	0.00000
	**Top 1,000**	**Bottom 1,000**	**Z-score**	***P*** **value**
Basin 1	4,493	1,979	37.196	0.00000
Basin 2	3,838	8	67.986	0.00000
Basin 3	2,534	1,000	28.166	0.00000
Basin 4	4,520	2,315	32.124	0.00000

Listed in the table are intervention success counts, *Z*-scores and *P* values for two-proportion *Z*-tests between the top 1,000 most frequent 3-templates and 1,000 random templates or the 1,000 least frequent templates. With 11 intervention starting states over 1,000 templates, each number of successes is out of 11,000 intervention attempts. In each case we find, with statistical confidence, the top frequency template group provides a higher proportion of successful interventions than the other groups.

#### Top templates for healthy and T-LGL attractors

We discuss two templates with high success rates leading to healthy attractor states followed by two templates with high rates of leading to T-LGL states. Lastly, we observe the single template which best differentiates the healthy and T-LGL states.

The first healthy intervention template of interest is for Basin 1, where we find a 3-template (Ceramide, PDGFR, and S1P set 0/1/0, respectively) with a high frequency and the ability to guide 73% of intervention attempts into Basin 1. In fact, the 27% remaining attempts not leading to Basin 1 lead to Basin 4, which is the other attractor classified as ‘healthy’. Thus, this template completely avoids the T-LGL basins. S1P is known to be 1 (*ON*) in a T-LGL state [36], and so it is biologically consistent to find it set to 0 (*OFF*) in the template.

The second healthy intervention template of interest is found in Basin 4 - the smallest classified basin considered in this study. In it, we find a powerful 1-template, TBET 0, which transitions 73% of intervention attempts to this very small basin covering only about 7% of the total state space. TBET is known to be 1 in T-LGL [34], so as a healthy basin, this setting is biologically consistent. Due to the small size of the basin, none of the top two or three templates were able to improve on this rate, and any that matched it included TBET 0 as part of the template. Any time a single variable can exert such a high degree of influence on a network, it is noteworthy.

The first T-LGL ‘intervention’ template is found in Basin 2. While it may seem contradictory to describe an ‘intervention’ which leads to a disease state, we nonetheless consider the power in the variable combination, perhaps as a trigger to avoid. In Basin 2, we quickly find the standout behavior of the three-template PDGFR/S1P/TBET, which is set 0/0/1, respectively. This template had a 100% intervention success rate, guiding network dynamics to Basin 2 upon every application. As in the previous template, we again see TBET; however, this time it is set 1, which is biologically consistent with the T-LGL state.

For Basin 3, which is also classified as a T-LGL attractor, we find another very powerful template, namely Ceramide, PDGFR, and S1P set 0/1/1, respectively. Not only does this template have a 100% intervention success rate but also each of its variable settings is known to be biologically consistent [36]–[38]. As the attractor state for Basin 3 is classified to be the stronger of the two T-LGL basins, and as Basin 3 is estimated to occupy only about 12% of the total state space, such a powerful template with perfect biological consistency is significant indeed.

Perhaps most interesting is the observation that this three-template in Basin 3 and the three-template for Basin 1 share the same three variables, with one going to 100% healthy attractors and the other going 100% to a T-LGL attractor. The templates have the same settings for Ceramide and PDGFR but differ in the setting for S1P, which is the biologically consistent setting across both basins. This reveals that, while Ceramide and PDFGR do not have biologically meaningful settings in Basin 1, for Basins 1 and 3, they still open the path, in terms of network dynamics, for the biologically consistent behavior of S1P to accurately and powerfully shift the network between healthy and T-LGL attractors. Thus, we conclude that, for this network, S1P is the key differentiator between healthy and T-LGL steady states, assisted by the combinatorial power of Ceramide and PDGFR.

Our work with the Leukemia network has produced some notable findings. First, we note that while all single-variable measures were unable to produce helpful intervention targets because of the 43 network variables, the template-based approaches did produce single and multi-variable intervention targets with observable separation in per-basin frequencies and in intervention success rates. Second, we observed that identifying the best template can benefit greatly from expert assignment of the basins of attraction to biological contexts (e.g., health vs. disease).

Finally, we saw that the biological significance of the results depends quite heavily on network rules. Basin 2 produced some biologically unexpected advice (i.e., S1P set to 0) within the templates, while the large, healthy Basin 1 and also the smaller T-LGL Basin 3 contained templates with immense power and great biological significance. This discrepancy can be attributed to many causes, including a network more focused on modeling the disease state (relatively rare) vs. the healthy state, which may be acceptable depending on the application. In our case, we saw two variables fixed between healthy and T-LGL interventions while the biologically consistent setting of the third variable, enabled by the combinatorial power of the first two, was able to dictate network fate.

Thus, while templates are capable of revealing novel biological insights, they may also reveal or confirm sensitivities in the network rule system that may or may not be desirable for a particular biological model. In the end, template-based analysis reveals the most powerful triggers for altering network dynamics into desired attractor basins strictly based upon the given Boolean rules. In our look into the T-LGL leukemia network, our templates were realized, and in most cases biologically reinforced, on a network with over 8 trillion states in the basin of attraction field based sampling only 50,000 initial states.

## Conclusions

Our work thus far has clearly established a usefulness in analyzing basins of attraction in identifying intervention targets. Our use of logic minimization reduces the representation of basins of attraction, and the template measures stratify the terms, revealing not only the key players in the system but also how to manipulate them. Perhaps the most important aspect of our revealed intervention targets is the fact that they are both basin- and value-specific; in other words, we provide not just targets, but how exactly to intervene (value) and also a context in which the intervention is appropriate (basin).

With small network sizes (less than 20), it is likely that many variables will either be important in some way (known beforehand) or may even represent an amalgamation of multiple entities. Thus, intervention targets revealed may be true, but they may also be obvious depending on the study. This, along with the fact that the single-variable measures fail in larger networks requiring a sampled state space, motivated our work to expand our approach for larger networks with dozens of variables and more, allowing us to include variables which are less well known and that may not be obvious intervention targets. By introducing the template counting approach to supersede the small network popularity and power measures, we have made possible the identification of powerful intervention targets despite sampled state spaces.

We first demonstrated the maintained success proportions of frequency-based template interventions between exhaustively enumerated and sampled state spaces. Convinced that key information was preserved by the measures despite sampling, we next showed the consistent success proportions across networks of increasing sizes as other methods fell away in performance. These investigations into robustness convinced us that the template approach was sure to provide the critical information needed regarding intervention targets.

We have also demonstrated the efficacy of the approach on a larger T-LGL leukemia network crafted by domain experts. We note that when all single-variable measures were unable to produce helpful intervention targets, the template-based approaches did produce single and multi-variable intervention targets with high intervention success rates. In the end, the template-based analysis revealed the most powerful triggers for altering network dynamics into desired attractor basins, and these results were realized, and in many cases, biologically corroborated, on a network with over 8 trillion states in the basin of attraction field based sampling only 50,000 initial states.

Despite the progress in sampling large state spaces, we will always be limited by the exponential growth of the state space with the number of variables. Fortunately as network sizes race into intractability, so too does the reliability of such networks, which is a direct influence on the quality of our results. In the end, our measures will always reveal the true triggers of network dynamics based on the given rules of the system. Thus, while they are capable of revealing novel biological insights, they may also reveal or confirm sensitivities in the network rule system that may or may not be desirable for a particular biological model. Since there are quality handmade networks with sizes into the dozens of variables, such as our T-LGL leukemia network, a Drosophila network from Albert et al. [6], and others, our leap to the 40 to 50 variable size level is significant. With improvements to algorithm implementation and with the incorporation of parallelization, we plan to improve the large networks approach in terms of speed and network size capability, ideally towards the 75 to 100 variable mark. At the same time, we also wish to incorporate the ability to prefer certain variables over others as template members if information regarding the downstream effects of intervention reveals possible redundancies. In addition, because interventions can and do alter the rule structure of the network, we wish to investigate the use of the PBN model, which is a stochastic extension of Boolean networks and is able to model such changes in biological context. In such cases, basins of attraction would need to be revised to reflect the new stochastic behavior, especially the steady-state distributions of PBNs, as these distributions reflect the long-run behavior of the network [13].

## Endnotes

^a^ We adhere to the traditional, synchronous update scheme due to its origins in relating attractors to biological cell types [9] and because its determinism is exploited by our analysis approach. Some validly claim that real biological systems do not ‘march in step’ and that asynchronous update mechanisms are more appropriate [25],[26]. Recent work [26],[35] comparing asynchronous update approaches identified the general asynchronous (GA) method, wherein a random node is updated at each time step, as superior. However, because neither do real biological systems ‘take turns’ updating, because synchronous networks are able to be analyzed by our methods without the dramatic reduction seen with asynchronous network analysis, because the nondeterminism associated with asynchronous networks may invalidate Kauffman’s hypothesis relating attractors to cell types, and because that synchronicity is still related to living systems [24], we work under the synchronous assumption even though there is no perfect answer.

^b^ Harmonic mean (*H*) is one kind of average. For two numbers, *x* and *y*, $H=\frac{2\text{xy}}{x+y}$.

^c^ Step 1 of this algorithm is a simple partitioning of the total iterations, and thus has a constant overhead. Step 2 of this algorithm is governed by the value *K*. In practice, *K* will be a small number and certainly much less than (and not dependent upon) *n*. Step 3 executes once for each unique template, namely, 2KnK times. It is known that the binomial coefficient nk is bounded above by (*n*×*e*/*k*)^*k*^[29]. Step 4 executes once for each term, where the number of terms is at most 2^*n*^ (i.e., no logic minimization at all). Steps 5 and 6 are a constant time operation. Because all steps are nested, the runtime is a product, from which constants can be removed, bounded above by a constant factor of 2^*n*^.

## Additional files

## Electronic supplementary material

Additional file 1:**Single-variable measures performance.** This supplement will show that variable popularity, variable power, and the harmonic mean of the two typically produce the most successful single variable intervention targets compared with other traditional network measures, including centralities, topological measures, etc. (PDF 236 KB)

Additional file 2:***P*** **value matrices for template simulation study.** This supplement contains pairwise *P* value matrices for sampled networks in the simulation study for template interventions. These values support the information communicated in Figures 4A,B. (XLSX 22 KB)

Additional file 3:**T-LGL leukemia network.** This supplement contains various additional information regarding the T-LGL Leukemia network. Specifically, this supplement will detail the steps and reasoning behind reducing the 60-variable T-LGL leukemia network down to 43 variables, the listing of Boolean network rules for the T-LGL leukemia network. These rules were translated directly into the Boolean functions governing the dynamics of the network, the Boolean states of the four main attractors with a description of their classification, and the listings of the 30 *F*-based templates for each of the four main basins of attraction with the highest intervention success estimates. The tables will also show the estimated chances of transitioning the network to each of the other three basins as well in order to illustrate how some intervention targets may be desirable for their ability to avoid undesirable basins in addition to their ability to find desirable ones. (PDF 210 KB)

Below are the links to the authors’ original submitted files for images.

Authors’ original file for figure 1

Authors’ original file for figure 2

Authors’ original file for figure 3

Authors’ original file for figure 4

Authors’ original file for figure 5

Authors’ original file for figure 6

## Abbreviations

*D* or TBDP: template basin distribution percentage
*F* or FREQ: frequency (based template)
HPP: harmonic mean of POP and POW
KxFREQ or KxTBDP: *F*- or *D*-based template with *x* variables (i.e., K1FREQ: K2TBDP, etc.)
POP: variable popularity
POW: variable power
