# Supplementary material for: Template-based intervention in Boolean network models of biological systems
Source: EURASIP J Bioinform Syst Biol. 2014 Jul 19;2014:11. doi: 10.1186/s13637-014-0011-4 (PMC5270454; doi:10.1186/s13637-014-0011-4)
Supplement: Supplementary file 1 — Additional file 1:Single-variable measures performance. This supplement will show that variable popularity, variable power, and the harmonic mean of the two typically produce the most successful single variable intervention targets compared with other traditional network measures, including centralities, topological measures, etc. (PDF 236 KB) [file 13637_2014_11_MOESM1_ESM.pdf]

Template-Based Intervention in Boolean Network Models of Biological Systems

Additional File 1: Single Variable Measures Performance

Michael P Verdicchio and Seungchan Kim

May 30, 2014

1 Robustness of Single Variable Measures

We claim that our small network measures of popularity and power identify targets for intervention in biological interaction networks modeled in the Boolean network framework. Furthermore we claim that the targets identified by our measures are better than those that can be identified by related approaches for identifying key network nodes, including two measures specific to Boolean networks. To justify this claim we present the following simulation experiment.

1.1 Problem Statement and Hypothesis

Are the popularity and power measures superior to related network measures for finding targets for intervention? We hypothesize that the popularity and power measures will lead to desired intervention outcomes with a greater rate of success than other methods and design the following experiment to test this prediction.

1.2 Experiment

Abstractly, an intervention should shift the steady behavior of a system to a different (usually more desirable) state. In the context of the Boolean network formalism, this is represented by shifting the steady behavior of the system, represented by an attractor state or cycle, into a different basin of attraction. A successful intervention needs only to shift the state from the starting attractor state into any state in the basin of the goal attractor, as the network dynamics will naturally bring the state to the attractor itself. Thus, we define a successful intervention as a single-variable modification to an attractor state which generates a state in the goal attractor basin.

1.2.1 Restrictions and Omitted Interventions

We make a few restrictions on our experiment to ensure a fair outlook on the results. First, attractor states are extremely stable and difficult to break out of with only a single variable perturbation. Nonetheless we restrict interventions to one single variable to induce a challenge and to simplify the experiment. Second, a “self-intervention” where the source and goal basins of attraction are the same could be considered a “maintenance” intervention if the basin is a desirable one; however this is a special case at best, and the robustness of attractor states will generate higher intervention success rates, inflating the overall performance. Thus we do not include interventions were the source and goal basins are the same, which also means that we discard networks with only one attractor basin. Third, our artificial intelligence planning work[2] showed that reaching the smallest, rarest basins often requires a series of interventions. Attempting these interventions with a single variable perturbation would unfairly bring scores down, so we omit interventions with a goal basin occupying less than 15% of the total state space.

1.2.2 Types of Interventions Performed

To create a simple comparison across a variety of approaches, we perform a single-variable perturbation upon the top variable candidate from each measure. If more than one variable shares the top rank for a particular measure, we choose between them randomly. If the measure includes a way to provide a value for the perturbation, we use the given value, otherwise we choose a value at random to be shared across any measure requiring one; in this way if more than one measure wishes to perturb the same variable but does not specify a value, they will all perturb the variable with the same random value so that no one method is advantaged within a particular instance.

We offer 14 methods for selecting an intervention target for comparison, with some of those being combinations of related measures. These methods are listed in Table 1 and are described here. First we offer our popularity and power measures. Empirical observations have shown us that sometimes the best variable can be identified by a collaboration between related measures. For example, let variable x have the highest popularity score (but a low power score), and let variable y have a high power score (but a low popularity score). Another variable z may not score higher in popularity or power than x or y, but it may have somewhat high values for both. In this case the harmonic mean of popularity and power may reveal z to be the top variable over x or y; thus we include the harmonic mean of popularity and power as the third way to identify an intervention target. These three measures are unique in that they have a specific value for each variable to be used in the intervention. This value used is based on the frequency of ON values in the reduced sum-of-product terms for the particular basin of attraction; if the frequency is greater than or equal to 0.5, a value of 1 is used, else, a value of 0 is used.

The next six methods for identifying an intervention target are variations

of the influence and sensitivity measures, which are designed specifically for Boolean networks[4]. We use influence, sensitivity, and their harmonic mean to identify the best target for intervention. Following, we use a basin-specific versions of influence and sensitivity (and their harmonic mean) to offer a basin-specific advantage akin to our popularity and power measures.

The remaining methods are related to the original network topology and common methods for identifying seemingly important network variables. First, we use the node with the highest degree, including both inbound and outbound edges, but not double-counting for self-regulation. Next, we use the nodes with the top centroid value, eccentricity value and betweenness centrality value[3]. We also include an intervention using a randomly chosen target for comparison purposes.

1.3 Methodology of the Experiment

We use Algorithm 1 to automate our experiments, repeating it for three different small network sizes. Each network is randomly generated using the biologically inspired connectivity probability distribution[1]. Rather than intervening upon randomly selected states, we intervene only upon attractor states. This is because we expect to find the system in a steady state, so it is the logical subset of states upon which to intervene. While some basins have a singleton attractor state, many basins have cyclic attractors with many states. To maximize the efficiency of the simulation, we will attempt an intervention from every attractor state in every basin in the state space to every other basin of significant size. This will allow us to attempt many interventions for each random network generated.

```
Input: Number of Variables, n; Number of Networks, nets
for i = 1 : nets do
    Generate Random Boolean Network BN(V, f) with n variables;
    Enumerate Basin of Attraction Field G(S, E);
    if Less than Two Attractor Basins then Discard Network,
    Continue;
    ;
    Compute all measures to identify intervention targets;
    foreach Basin B_i ∈ B as the SOURCE do
        foreach Attractor State a_i ∈ A_i ∈ B_i do
            foreach Basin B_j ≠ B_i as the GOAL do
                if B_j occupies < 15% of B then Discard Intervention,
                Continue;
                ;
                foreach Intervention Target Method do
                    Select one top intervention target;
                    Select or generate value for target;
                    Apply intervention to a_i to produce new state x_k;
                    Identify DESTINATION basin B_k such that
                    x_k ∈ A_k ∈ B_k;
                    if B_j == B_k then
                        Record Success;
                    else
                        Record Failure;
```

Algorithm 1: Algorithm for Small Network Measures Comparison: The *Continue* statement indicates skipping the remainder of the specified loop's current iteration and continuing the same loop at the next iteration.

We apply Algorithm 1 with the following input parameters: $n = \{7, 8, 9, 10, 11, 12, 13, 14, 15, 16\}$, and $nets = 400$ for each network size. Due to the varying numbers of attractor basins as well as the varying lengths of attractor cycles, these input combinations resulted in 95,870 attempted interventions.

1.4 Results of Experiment

Overall we observed the dominance of the popularity and power measures (along with their harmonic mean) in identifying targets for intervention over other strategies, which is shown in Table 1 and in Fig. 1. The requested interventions were increasingly difficult with network size due to their restriction to a single variable. The benefits of counting easy "self-interventions" (i.e. staying in the same basin) were not included, and the detriments of attempting interventions to very rare basins were also omitted to get a fair idea of performance. The influence measure showed the best behavior after the popularity and power measures, and the topological measures performed poorly.

On the smaller networks, our measures dominate with a great deal of separation in performance. As the network size increases, however, the performance of the various methods begins to converge. This is the primary motivation for

our introduction of template based approaches. We note that besides our methods, the Boolean-network-specific measure of influence (and it's basin-specific counterpart) perform the best on average of the related measures.

Sensitivity is a measure such that variables with low sensitivity can maintain their states better than others with higher sensitivity[5]. However this attribute does not imply the potential for regulatory control like the influence measure does. Thus it is not surprising to see influence dominate the sensitivity measure. It has been said that a variable with a low sensitivity but a high influence would make the best intervention targets[5]; however while our results certainly shown an improvement over sensitivity alone by the harmonic mean of influence and (low) sensitivity, the combination of the two was not able to outperform influence alone in either the whole-network or basin-specific cases. Of the topological measures, only node degree and betweenness passed, on average, the performance of a random intervention target. Centroid and eccentricity failed completely, oftentimes being outperformed by a random intervention.

Intervention success counts and numbers of trials were used to calculate confidence intervals according to the Binomial test, since our interventions qualified as Bernoulli trials. While we can visually see separation in the figure and elevated average and individual performances, the confidence intervals of our small network measures did indeed overlap with some of the best scoring related measures. But, while topological measures can certainly lend insight at the top level of networks, the underlying complexities of the Boolean model are clearly better elucidated by measures designed specifically for the Boolean model. Of those, the robustness of the popularity and power measures is clearly demonstrated, despite their ability to statistically separate in the given trial. We hypothesize that if the interventions were to begin from random states and not just from attractor states that we would gain the separation needed for a statistical significance; however our motivation to move to larger networks and introduce template based approaches renders this point moot.

1.5 Conclusions

We believe the dominating success of popularity and power measures is due to the inherent inclusion of a specific value to use in the intervention, and that both target variables and their values are specific to the goal basin. In each intervention instance, both the target and the value were chosen from the popularity and power measures specific to the goal basin. These advantages absent in other methods clearly resulted in the higher performance. It is also not surprising to see the influence measures appearing next in the performance after our measures for two reasons. First, like our measures, it is a measure designed in and thus taking advantage of the Boolean network model. Second, the basin-specific influence we designed to add a degree of fairness demonstrated a benefit from that modification. The topological measures without either basin or value specificity naturally performed the most poorly.

Despite a lack of complete separation of confidence intervals, but based on the consistent top performance of the popularity and power measures across

Table 1: Single Variable Network Measures Compared: We have shown the ability of the single variable measures of popularity and power to identify intervention targets missed by other related measures. In this table we report average success estimates for intervening with a single variable chosen by a variety of methods and their combinations. Random networks with between 7 and 16 variables were generated and those networks with between 2 and 7 basins of attraction were intervened upon. Overall, we observed popularity, power, and their harmonic mean as supplying the most effective intervention targets. After these, the Boolean-network-specific measures of influence and sensitivity performed best, especially their basin-specific versions invented for this study. Thus we conclude that popularity, power and the harmonic mean of popularity and power produce the best single variable intervention targets.

**Variables:**	**7**	**8**	**9**	**10**	**11**	**12**	**13**	**14**	**15**	**16**	**AVG**
Popularity	0.297	0.325	0.296	0.302	0.292	0.292	0.289	0.318	0.312	0.314	0.304
Power	0.315	0.311	0.289	0.281	0.286	0.294	0.277	0.314	0.320	0.314	0.300
$H(p(v), P_V(v))$	0.295	0.317	0.288	0.299	0.291	0.293	0.281	0.314	0.314	0.314	0.301
Influence	0.266	0.281	0.278	0.285	0.280	0.276	0.292	0.299	0.307	0.314	0.288
Sensitivity	0.250	0.265	0.265	0.250	0.245	0.275	0.249	0.268	0.275	0.276	0.262
$H(Infl., Sens.)$	0.272	0.276	0.273	0.287	0.272	0.277	0.284	0.274	0.304	0.310	0.283
Basin Influence	0.280	0.273	0.276	0.283	0.278	0.277	0.291	0.298	0.307	0.314	0.288
Basin Sensitivity	0.251	0.272	0.257	0.249	0.241	0.272	0.246	0.271	0.279	0.272	0.261
$H(B. Infl., B. Sens.)$	0.260	0.278	0.273	0.285	0.269	0.279	0.284	0.273	0.299	0.303	0.280
Degree	0.262	0.267	0.270	0.250	0.269	0.283	0.263	0.285	0.297	0.306	0.275
Centroid	0.234	0.244	0.262	0.230	0.233	0.262	0.242	0.252	0.253	0.298	0.251
Eccentricity	0.235	0.246	0.255	0.221	0.239	0.273	0.237	0.256	0.256	0.293	0.251
Betweenness	0.258	0.274	0.269	0.253	0.276	0.283	0.273	0.285	0.289	0.309	0.277
Random	0.254	0.264	0.255	0.237	0.247	0.280	0.252	0.254	0.277	0.304	0.262

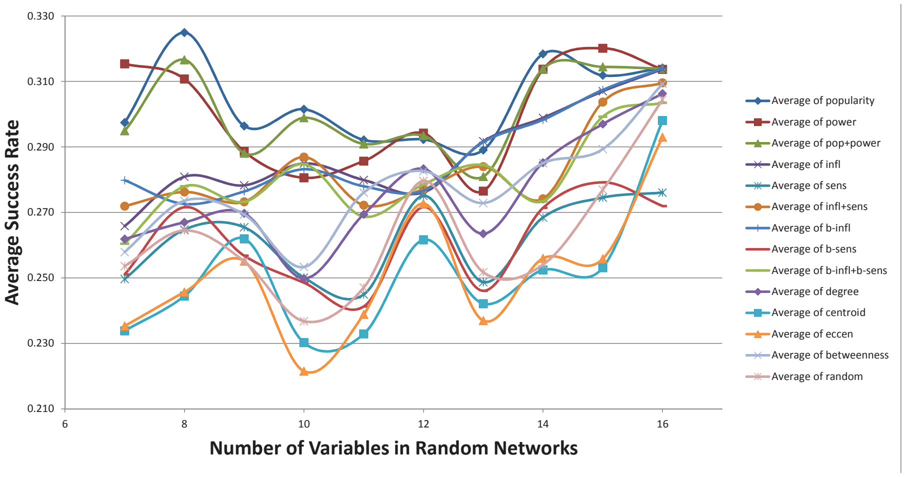

Figure 1: Small Network Robustness Experiment Results: Here we see a graphical representation of Table 1. We observe the dominance of popularity, power, and their harmonic mean over other related measures..

various network sizes and over thousands of interventions, we fail to reject our hypothesis and conclude that the popularity and power measures lead to desired intervention outcomes with a greater rate of success than other topological, centrality-based, and even Boolean network specific methods.

References

[1] Reka Albert and Hans G. Othmer. The topology of the regulatory interactions predicts the expression pattern of the segment polarity genes in drosophila melanogaster. *Journal of Theoretical Biology*, 223(1):1–18, July 2003.

[2] Daniel Bryce, Michael P. Verdicchio, and Seungchan Kim. Planning interventions in biological networks. *ACM Transactions on Intelligent Systems and Technology*, To Appear.

[3] Giovanni Scardoni, Michele Petterlini, and Carlo Laudanna. Analyzing biological network parameters with centiscape. *Bioinformatics*, 25(21):2857–2859, November 2009.

[4] I. Shmulevich, E. R. Dougherty, and Wei Zhang. From boolean to probabilistic boolean networks as models of genetic regulatory networks. *Proceedings of the IEEE*, 90(11):1778–1792, 2002.

[5] Ilya Shmulevich, Edward R. Dougherty, Seungchan Kim, and Wei Zhang. Probabilistic boolean networks: a rule-based uncertainty model for gene regulatory networks. *Bioinformatics*, 18(2):261–274, February 2002.
