# Supplementary material for: Template-based intervention in Boolean network models of biological systems
Source: EURASIP J Bioinform Syst Biol. 2014 Jul 19;2014:11. doi: 10.1186/s13637-014-0011-4 (PMC5270454; doi:10.1186/s13637-014-0011-4)
Supplement: Supplementary file 3 — Additional file 3:T-LGL leukemia network. This supplement contains various additional information regarding the T-LGL Leukemia network. Specifically, this supplement will detail the steps and reasoning behind reducing the 60-variable T-LGL leukemia network down to 43 variables, the listing of Boolean network rules for the T-LGL leukemia network. These rules were translated directly into the Boolean functions governing the dynamics of the network, the Boolean states of the four main attractors with a description of their classification, and the listings of the 30 F-based templates for each of the four main basins of attraction with the highest intervention success estimates. The tables will also show the estimated chances of transitioning the network to each of the other three basins as well in order to illustrate how some intervention targets may be desirable for their ability to avoid undesirable basins in addition to their ability to find desirable ones. (PDF 210 KB) [file 13637_2014_11_MOESM3_ESM.pdf]

Template-Based Intervention in Boolean Network Models of Biological Systems

Additional file 3: Additional Information for T-LGL Leukemia Network

Michael P Verdicchio and Seungchan Kim

May 20, 2014

This supplement provides additional information for the T-LGL Leukemia network.

1 Network Simplification

The original network [3] created from the literature contained 128 nodes and 287 edges, but was simplified by software and manual adjustments to 60 nodes and 142 regulatory edges. In order to comply with current algorithmic implementations we were required to further reduce this network to have less than 53 variables; this was due to a dependency on storing information in a double-precision 64-bit variable only capable of storing unsigned integers up to $2^{53} - 1$. Our future work includes updating the software around this limitation, which is entirely possible with the use of variable precision integers.

After collaborating with Dr. Réka Albert, a principle author from [3], we performed the following reductions on the network according to techniques described in related work involving this network [1, 2]. The goal of the reduction is twofold. First, we wish to remove nodes which mask the dynamic behavior of the network variables (i.e. the overarching influence of Apoptosis as well as root nodes); since steady-state analysis will be performed over many randomly generated states, control variables are not necessary since variables they control will be forced to take on different values through random starting state assignment. Second we wish to reduce the size of the network to less than 53 nodes in order to work with the current algorithm implementations. After the simplifications described below we obtain the 43-variable version shown in Fig. 5 in the main manuscript.

1. Delete all references to Apoptosis. Since it can turn off every cell it dominates the Boolean logic and focuses the study on the stationary states of the living cell.

2. Delete all root nodes: Stimuli, IL15, PDGF, CD45, TAX, Stimuli2 and P2. P2 becomes root after deleting Stimuli2.; These control nodes emerge as the dominant variables upon network analysis. Removing them reveals which OTHER variables are powerful and may be targets for intervention.

3. Delete node A20. A20 has only one input edge and one output edge forming a closed loop from NFKB to A20 to TRADD and back to NFKB. Replace A20 in the rule for TRADD with the same value of NFKB because the rule for A20 is A20 = NFKB.

4. Delete node CTLA4. CTLA4 has only one input and one output edge and forms a simple loop with TCR.

5. Delete TCR. After deleting CTLA4, and Stimuli, TCR has no inputs.

6. Delete GPCR and SMAD. The chain goes from S1P, to GPCR, to SMAD, to IFNG. GPCR and SMAD have one input and one output, so connect S1P to IFNG, replacing SMAD in the rule.

7. Delete MEK, which has only 1 input and 1 output. Connect RAS directly to ERK instead of through MEK.

8. Delete P27. Delete it from the rule in Proliferation as well. Proliferation already has input from STAT3, which is P27's parent.

9. Delete RANTES. Delete it from the rule in JAK as well. JAK already has input from NFKB, which is RANTES's parent.

10. Delete SPHK1. Connect PDGFR directly to S1P1 creating a direct loop instead of one interrupted by SPHK1.

2 Boolean Rules

Boolean rules after simplification are shown in Table 4.

3 Attractors

Identified attractors are shown in Table 4.

4 Top Templates Results

With reference to the methodology and T-LGL results section of the main paper, here we summarize the top thirty most frequent intervention templates from each template size from each of the four main basins in the network. To estimate a success rate, we apply each top template intervention to all eleven attractor states across all basins in the T-LGL network and compute the distribution of basins reached. If a template causes a significant number of these

attractor states to jump to (or remain in) a desired basin, such a template is of great interest. In general we expect an attractor state to remain robust to perturbation but expect the larger templates to have the best chance at changing the steady state of the system. Since Basins 1 and 4 each contain a 4-cycle of attractor states, we expect at least 4 of the 11 destination states (36%) to remain in those basins (due to the expectation of robustness mentioned). Likewise the remaining basins each have a single attractor state each and we expect 1 of the 11 destination states (9%) to remain in these basins. Of the top 50 templates for each k and each basin, we indeed saw these expected distributions of goal states very frequently. Numbers above and beyond these expectations warrant closer inspection, which we provide for the most interesting templates listed in Table 4 in the main manuscript. Templates listed in Table 4 and discussed in the manuscript results are highlighted with horizontal rule lines:

- Basin 1, 3-Templates, Rank 18, Table 4
- Basin 2, 3-Templates, Rank 27, Table 4
- Basin 3, 3-Templates, Rank 1, Table 4
- Basin 4, 1-Templates, Rank 6, Table 4

Table 1: Boolean Rules for T-LGL Leukemia Network: After network simplification from Zhang *et al.*[3] based on techniques from Saadatpour *et al.*[1, 2], the following update rules were formed. The first column represents the next state of the listed variables and the second column refers to the current state of the variable(s). $\wedge$, $\vee$ and $\neg$ represent logical AND, OR and NOT, respectively.

Variable	Boolean Function
BID	(Caspase $\vee$ GZMB) $\wedge$ $\neg$ (BclxL $\vee$ MCL1)
BclxL	(NFKB $\vee$ STAT3) $\wedge$ $\neg$ (BID $\vee$ GZMB $\vee$ DISC)
CREB	(ERK $\wedge$ IFNG)
Caspase	((((TRADD $\vee$ GZMB) $\wedge$ BID) $\wedge$ $\neg$ IAP) $\vee$ DISC)
Ceramide	Fas $\wedge$ $\neg$ S1P
Cytoskeleton_signaling	FYN
DISC	(FasT $\wedge$ ((Fas $\wedge$ IL2) $\vee$ Ceramide $\vee$ (Fas $\wedge$ $\neg$ FLIP)))
ERK	(RAS $\wedge$ PI3K)
FLIP	(NFKB $\vee$ (CREB $\wedge$ IFNG)) $\wedge$ $\neg$ DISC
FYN	IL2RB
Fas	(FasT $\wedge$ FasL) $\wedge$ $\neg$ SFas
FasL	(STAT3 $\vee$ NFKB $\vee$ NFAT $\vee$ ERK)
FasT	NFKB
GAP	(RAS $\vee$ (PDGFR $\wedge$ GAP)) $\wedge$ $\neg$ IL2
GRB2	(IL2RB $\vee$ ZAP70)
GZMB	((CREB $\wedge$ IFNG) $\vee$ TBET)
IAP	NFKB $\wedge$ $\neg$ BID
IFNG	(IL2 $\wedge$ IFNGT) $\wedge$ $\neg$ S1P
IFNGT	(TBET $\vee$ STAT3 $\vee$ NFAT)
IL2	(NFKB $\vee$ STAT3 $\vee$ NFAT) $\wedge$ $\neg$ TBET
IL2RA	(IL2 $\wedge$ IL2RAT) $\wedge$ $\neg$ IL2RA
IL2RAT	(IL2 $\wedge$ (STAT3 $\vee$ NFKB))
IL2RB	IL2RBT $\wedge$ IL2
IL2RBT	(ERK $\wedge$ TBET)
JAK	(IL2RA $\vee$ IL2RB $\vee$ IFNG) $\wedge$ $\neg$ SOCS
LCK	IL2RB $\wedge$ $\neg$ ZAP70
MCL1	MCL1 = (IL2RB $\wedge$ STAT3 $\wedge$ NFKB $\wedge$ PI3K) $\wedge$ $\neg$ DISC
NFAT	PI3K
NFKB	((TPL2 $\vee$ PI3K) $\vee$ (FLIP $\wedge$ TRADD $\wedge$ IAP))
PDGFR	S1P
PI3K	(PDGFR $\vee$ RAS)
PLCG1	(GRB2 $\vee$ PDGFR)
Proliferation	STAT3
RAS	(GRB2 $\vee$ PLCG1) $\wedge$ $\neg$ GAP
S1P	PDGFR $\wedge$ $\neg$ Ceramide
SFas	FasT $\wedge$ S1P
SOCS	JAK $\wedge$ $\neg$ IL2
STAT3	JAK
TBET	(JAK $\vee$ TBET)
TNF	NFKB
TPL2	PI3K $\wedge$ TNF
TRADD	TNF $\wedge$ $\neg$ (IAP $\vee$ NFKB)
ZAP70	LCK $\wedge$ $\neg$ FYN

Table 2: Attractor States for T-LGL Leukemia Network: The four basins are classified as either Normal (i.e. healthy, normal apoptosis function) or T-LGL (i.e. cancer state) based on the values of certain key variables in the steady attractor states/cycles.

Variable	Basin 1				Basin 4				Basin 2	Basin 3
BID	1	1	1	1	1	1	1	1	1	1
BclxL	0	0	0	0	0	0	0	0	0	0
CREB	0	0	0	0	0	0	0	0	0	0
Caspase	1	1	1	1	1	1	1	1	1	1
Ceramide	0	1	0	1	1	0	1	0	0	0
Cytoskeleton_signaling	0	0	0	0	0	0	0	0	0	0
DISC	1	0	1	0	1	1	1	1	0	0
ERK	0	0	0	0	0	0	0	0	0	0
FLIP	1	0	1	0	0	0	0	0	0	1
FYN	0	0	0	0	0	0	0	0	0	0
Fas	1	0	1	0	0	1	0	1	0	0
FasL	1	1	1	1	1	1	1	1	0	1
FasT	1	1	1	1	1	1	1	1	0	1
GAP	0	1	0	0	0	0	1	0	0	1
GRB2	0	0	0	0	0	0	0	0	0	0
GZMB	1	1	1	1	1	1	1	1	1	1
IAP	0	0	0	0	0	0	0	0	0	0
IFNG	0	0	0	0	0	0	0	0	0	0
IFNGT	1	1	1	1	1	1	1	1	1	1
IL2	0	0	0	0	0	0	0	0	0	0
IL2RA	0	0	0	0	0	0	0	0	0	0
IL2RAT	0	0	0	0	0	0	0	0	0	0
IL2RB	0	0	0	0	0	0	0	0	0	0
IL2RBT	0	0	0	0	0	0	0	0	0	0
JAK	0	0	0	0	0	0	0	0	0	0
LCK	0	0	0	0	0	0	0	0	0	0
MCL1	0	0	0	0	0	0	0	0	0	0
NFAT	1	0	1	0	0	1	0	1	0	1
NFKB	1	1	1	1	1	1	1	1	0	1
PDGFR	1	0	1	0	0	1	0	1	0	1
PI3K	0	1	0	1	1	0	1	0	0	1
PLCG1	0	1	0	1	1	0	1	0	0	1
Proliferation	0	0	0	0	0	0	0	0	0	0
RAS	1	0	0	0	0	1	0	0	0	0
S1P	0	1	0	1	1	0	1	0	0	1
SFas	1	0	1	0	0	1	0	1	0	1
SOCS	0	0	0	0	0	0	0	0	0	0
STAT3	0	0	0	0	0	0	0	0	0	0
TBET	1	1	1	1	1	1	1	1	1	1
TNF	1	1	1	1	1	1	1	1	0	1
TPL2	1	0	1	0	0	1	0	1	0	1
TRADD	0	0	0	0	0	0	0	0	0	0
ZAP70	0	0	0	0	0	0	0	0	0	0

Table 3: Top 30 1-Templates for Leukemia Network Basin 1

RANK	Variable	Value	FREQ	SUCCESS	B1	B2	B3	B4	B5
1	MCL1	0	37204	0.36	0.36	0.09	0.09	0.36	0.09
2	FasL	1	35721	0.36	0.36	0.09	0.09	0.36	0.09
3	TBET	1	35405	0.36	0.36	0.18	0.09	0.36	0.00
4	IFNGT	1	34977	0.36	0.36	0.09	0.09	0.36	0.09
5	BclxL	0	34861	0.36	0.36	0.09	0.09	0.36	0.09
6	Ceramide	0	34861	0.36	0.36	0.09	0.09	0.36	0.09
7	TRADD	0	34840	0.36	0.36	0.09	0.09	0.36	0.09
8	IFNG	0	34792	0.36	0.36	0.09	0.09	0.36	0.09
9	IL2RA	0	33928	0.36	0.36	0.09	0.09	0.36	0.09
10	Fas	0	33697	0.55	0.55	0.09	0.09	0.18	0.09
11	ZAP70	0	33070	0.36	0.36	0.09	0.09	0.36	0.09
12	IL2RB	0	32936	0.36	0.36	0.09	0.09	0.36	0.09
13	CREB	0	32750	0.36	0.36	0.09	0.09	0.36	0.09
14	PLCG1	1	31966	0.36	0.36	0.18	0.09	0.36	0.00
15	LCK	0	31537	0.36	0.36	0.09	0.09	0.36	0.09
16	SOCS	0	31438	0.36	0.36	0.09	0.09	0.36	0.09
17	NFKB	1	31197	0.36	0.36	0.09	0.09	0.36	0.09
18	ERK	0	30968	0.36	0.36	0.09	0.09	0.36	0.09
19	TPL2	0	30842	0.55	0.55	0.09	0.09	0.18	0.09
20	DISC	0	30814	0.55	0.55	0.09	0.09	0.18	0.09
21	PI3K	1	30798	0.36	0.36	0.18	0.09	0.36	0.00
22	IL2RBT	0	30794	0.36	0.36	0.09	0.09	0.36	0.09
23	GAP	0	30120	0.36	0.36	0.09	0.09	0.36	0.09
24	GZMB	1	29936	0.36	0.36	0.09	0.09	0.36	0.09
25	IAP	0	29855	0.36	0.36	0.09	0.09	0.36	0.09
26	IL2	0	29322	0.36	0.36	0.09	0.09	0.36	0.09
27	GRB2	1	29180	0.36	0.36	0.18	0.09	0.36	0.00
28	SFas	0	29023	0.36	0.36	0.09	0.09	0.36	0.09
29	BID	0	28779	0.36	0.36	0.09	0.09	0.36	0.09
30	PDGFR	1	28268	0.45	0.45	0.00	0.09	0.45	0.00

Table 4: Top 30 1-Templates for Leukemia Network Basin 2

RANK	Variable	Value	FREQ	SUCCESS	B1	B2	B3	B4	B5
1	S1P	0	46989	0.45	0.27	0.45	0.00	0.18	0.09
2	PDGFR	0	40833	0.45	0.27	0.45	0.00	0.18	0.09
3	SFas	0	37512	0.09	0.36	0.09	0.09	0.36	0.09
4	MCL1	0	36675	0.09	0.36	0.09	0.09	0.36	0.09
5	FasL	1	35071	0.09	0.36	0.09	0.09	0.36	0.09
6	TRADD	0	34577	0.09	0.36	0.09	0.09	0.36	0.09
7	BclxL	0	33922	0.09	0.36	0.09	0.09	0.36	0.09
8	IFNGT	1	33405	0.09	0.36	0.09	0.09	0.36	0.09
9	ZAP70	0	33168	0.09	0.36	0.09	0.09	0.36	0.09
10	IL2RB	0	32959	0.09	0.36	0.09	0.09	0.36	0.09
11	IL2RA	0	32580	0.09	0.36	0.09	0.09	0.36	0.09
12	CREB	0	31818	0.09	0.36	0.09	0.09	0.36	0.09
13	IL2RBT	0	31236	0.09	0.36	0.09	0.09	0.36	0.09
14	SOCS	0	31202	0.09	0.36	0.09	0.09	0.36	0.09
15	TBET	1	31043	0.18	0.36	0.18	0.09	0.36	0.00
16	LCK	0	30996	0.09	0.36	0.09	0.09	0.36	0.09
17	NFKB	1	30689	0.09	0.36	0.09	0.09	0.36	0.09
18	GAP	0	30321	0.09	0.36	0.09	0.09	0.36	0.09
19	ERK	0	30080	0.09	0.36	0.09	0.09	0.36	0.09
20	TPL2	0	29021	0.09	0.55	0.09	0.09	0.18	0.09
21	Fas	0	28701	0.09	0.55	0.09	0.09	0.18	0.09
22	IFNG	0	28414	0.09	0.36	0.09	0.09	0.36	0.09
23	IAP	0	27628	0.09	0.36	0.09	0.09	0.36	0.09
24	GZMB	1	27597	0.09	0.36	0.09	0.09	0.36	0.09
25	PLCG1	1	27306	0.18	0.36	0.18	0.09	0.36	0.00
26	FLIP	0	27280	0.09	0.18	0.09	0.09	0.55	0.09
27	IL2	0	27011	0.09	0.36	0.09	0.09	0.36	0.09
28	GRB2	1	26652	0.18	0.36	0.18	0.09	0.36	0.00
29	BID	0	26610	0.09	0.36	0.09	0.09	0.36	0.09
30	DISC	0	26371	0.09	0.55	0.09	0.09	0.18	0.09

Table 5: Top 30 1-Templates for Leukemia Network Basin 3

RANK	Variable	Value	FREQ	SUCCESS	B1	B2	B3	B4	B5
1	Ceramide	0	14404	0.09	0.36	0.09	0.09	0.36	0.09
2	PDGFR	1	14404	0.09	0.45	0.00	0.09	0.45	0.00
3	S1P	1	14404	0.45	0.27	0.00	0.45	0.27	0.00
4	IFNG	0	11323	0.09	0.36	0.09	0.09	0.36	0.09
5	PLCG1	1	11278	0.09	0.36	0.18	0.09	0.36	0.00
6	PI3K	1	11256	0.09	0.36	0.18	0.09	0.36	0.00
7	MCL1	0	11018	0.09	0.36	0.09	0.09	0.36	0.09
8	FasL	1	10664	0.09	0.36	0.09	0.09	0.36	0.09
9	TRADD	0	10376	0.09	0.36	0.09	0.09	0.36	0.09
10	BclxL	0	10289	0.09	0.36	0.09	0.09	0.36	0.09
11	IFNGT	1	10043	0.09	0.36	0.09	0.09	0.36	0.09
12	IL2RA	0	9994	0.09	0.36	0.09	0.09	0.36	0.09
13	DISC	0	9927	0.09	0.55	0.09	0.09	0.18	0.09
14	NFKB	1	9911	0.09	0.36	0.09	0.09	0.36	0.09
15	Fas	0	9867	0.09	0.55	0.09	0.09	0.18	0.09
16	ZAP70	0	9785	0.09	0.36	0.09	0.09	0.36	0.09
17	IL2RB	0	9748	0.09	0.36	0.09	0.09	0.36	0.09
18	CREB	0	9733	0.09	0.36	0.09	0.09	0.36	0.09
19	TBET	1	9389	0.09	0.36	0.18	0.09	0.36	0.00
20	SOCS	0	9286	0.09	0.36	0.09	0.09	0.36	0.09
21	LCK	0	9270	0.09	0.36	0.09	0.09	0.36	0.09
22	IL2RBT	0	9187	0.09	0.36	0.09	0.09	0.36	0.09
23	ERK	0	8662	0.09	0.36	0.09	0.09	0.36	0.09
24	IAP	0	8640	0.09	0.36	0.09	0.09	0.36	0.09
25	GRB2	1	8483	0.09	0.36	0.18	0.09	0.36	0.00
26	GAP	0	8439	0.09	0.36	0.09	0.09	0.36	0.09
27	TPL2	0	8436	0.09	0.55	0.09	0.09	0.18	0.09
28	GZMB	1	8355	0.09	0.36	0.09	0.09	0.36	0.09
29	BID	0	8341	0.09	0.36	0.09	0.09	0.36	0.09
30	NFAT	1	8244	0.09	0.36	0.18	0.09	0.36	0.00

Table 6: Top 30 1-Templates for Leukemia Network Basin 4

RANK	Variable	Value	FREQ	SUCCESS	B1	B2	B3	B4	B5
1	NFKB	1	7260	0.36	0.36	0.09	0.09	0.36	0.09
2	FasL	1	6735	0.36	0.36	0.09	0.09	0.36	0.09
3	MCL1	0	6594	0.36	0.36	0.09	0.09	0.36	0.09
4	PI3K	1	6428	0.36	0.36	0.18	0.09	0.36	0.00
5	TRADD	0	6417	0.36	0.36	0.09	0.09	0.36	0.09
6	TBET	0	6404	0.73	0.00	0.00	0.09	0.73	0.18
7	IL2RBT	0	6342	0.36	0.36	0.09	0.09	0.36	0.09
8	JAK	0	6318	0.36	0.36	0.09	0.09	0.36	0.09
9	FasT	1	6212	0.36	0.36	0.09	0.09	0.36	0.09
10	IL2RB	0	6091	0.36	0.36	0.09	0.09	0.36	0.09
11	BclxL	0	5955	0.36	0.36	0.09	0.09	0.36	0.09
12	IFNG	0	5941	0.36	0.36	0.09	0.09	0.36	0.09
13	ZAP70	0	5928	0.36	0.36	0.09	0.09	0.36	0.09
14	TNF	1	5925	0.36	0.36	0.09	0.09	0.36	0.09
15	CREB	0	5888	0.36	0.36	0.09	0.09	0.36	0.09
16	PLCG1	1	5845	0.36	0.36	0.18	0.09	0.36	0.00
17	SOCS	0	5758	0.36	0.36	0.09	0.09	0.36	0.09
18	STAT3	0	5756	0.36	0.36	0.09	0.09	0.36	0.09
19	IL2RA	0	5737	0.36	0.36	0.09	0.09	0.36	0.09
20	LCK	0	5720	0.36	0.36	0.09	0.09	0.36	0.09
21	Ceramide	0	5622	0.36	0.36	0.09	0.09	0.36	0.09
22	GAP	0	5474	0.36	0.36	0.09	0.09	0.36	0.09
23	IFNGT	1	5397	0.36	0.36	0.09	0.09	0.36	0.09
24	IL2	1	5356	0.55	0.18	0.09	0.09	0.55	0.09
25	NFAT	1	5253	0.36	0.36	0.18	0.09	0.36	0.00
26	GZMB	0	5101	0.36	0.36	0.09	0.09	0.36	0.09
27	SFas	0	4965	0.36	0.36	0.09	0.09	0.36	0.09
28	PDGFR	1	4898	0.45	0.45	0.00	0.09	0.45	0.00
29	Proliferation	0	4849	0.36	0.36	0.09	0.09	0.36	0.09
30	BID	0	4840	0.36	0.36	0.09	0.09	0.36	0.09

Table 7: Top 30 2-Templates for Leukemia Network Basin 1

RANK	Variables		Values		FREQ	SUCCESS	B1	B2	B3	B4	B5
1	FasL	MCL1	1	0	29352	0.36	0.36	0.09	0.09	0.36	0.09
2	BclxL	MCL1	0	0	28755	0.36	0.36	0.09	0.09	0.36	0.09
3	IFNG	MCL1	0	0	28511	0.36	0.36	0.09	0.09	0.36	0.09
4	Ceramide	MCL1	0	0	28485	0.36	0.36	0.09	0.09	0.36	0.09
5	IFNGT	MCL1	1	0	28412	0.36	0.36	0.09	0.09	0.36	0.09
6	MCL1	TBET	0	1	28410	0.36	0.36	0.18	0.09	0.36	0.00
7	MCL1	TRADD	0	0	28348	0.36	0.36	0.09	0.09	0.36	0.09
8	FasL	IFNGT	1	1	28311	0.36	0.36	0.09	0.09	0.36	0.09
9	IL2RA	MCL1	0	0	27759	0.36	0.36	0.09	0.09	0.36	0.09
10	FasL	TRADD	1	0	27617	0.36	0.36	0.09	0.09	0.36	0.09
11	FasL	TBET	1	1	27444	0.36	0.36	0.18	0.09	0.36	0.00
12	Ceramide	FasL	0	1	27420	0.36	0.36	0.09	0.09	0.36	0.09
13	Fas	MCL1	0	0	27402	0.55	0.55	0.09	0.09	0.18	0.09
14	FasL	IFNG	1	0	27354	0.36	0.36	0.09	0.09	0.36	0.09
15	IFNGT	TBET	1	1	27268	0.36	0.36	0.18	0.09	0.36	0.00
16	Ceramide	IFNG	0	0	27028	0.36	0.36	0.09	0.09	0.36	0.09
17	BclxL	FasL	0	1	26988	0.36	0.36	0.09	0.09	0.36	0.09
18	IFNG	IFNGT	0	1	26865	0.36	0.36	0.09	0.09	0.36	0.09
19	MCL1	ZAP70	0	0	26861	0.36	0.36	0.09	0.09	0.36	0.09
20	BclxL	IFNG	0	0	26845	0.36	0.36	0.09	0.09	0.36	0.09
21	IL2RB	MCL1	0	0	26696	0.36	0.36	0.09	0.09	0.36	0.09
22	IFNGT	TRADD	1	0	26678	0.36	0.36	0.09	0.09	0.36	0.09
23	TBET	TRADD	1	0	26619	0.36	0.36	0.18	0.09	0.36	0.00
24	Ceramide	TRADD	0	0	26591	0.36	0.36	0.09	0.09	0.36	0.09
25	Ceramide	TBET	0	1	26568	0.36	0.36	0.18	0.09	0.36	0.00
26	FasL	IL2RA	1	0	26532	0.36	0.36	0.09	0.09	0.36	0.09
27	IFNG	TBET	0	1	26530	0.36	0.36	0.18	0.09	0.36	0.00
28	Ceramide	IFNGT	0	1	26512	0.36	0.36	0.09	0.09	0.36	0.09
29	CREB	MCL1	0	0	26439	0.36	0.36	0.09	0.09	0.36	0.09
30	IFNG	TRADD	0	0	26428	0.36	0.36	0.09	0.09	0.36	0.09

Table 8: Top 30 2-Templates for Leukemia Network Basin 2

RANK	Variables		Values		FREQ	SUCCESS	B1	B2	B3	B4	B5
1	PDGFR	S1P	0	0	40833	0.91	0.00	0.91	0.00	0.00	0.09
2	S1P	SFas	0	0	37512	0.45	0.27	0.45	0.00	0.18	0.09
3	MCL1	S1P	0	0	36675	0.45	0.27	0.45	0.00	0.18	0.09
4	FasL	S1P	1	0	35071	0.45	0.27	0.45	0.00	0.18	0.09
5	S1P	TRADD	0	0	34577	0.45	0.27	0.45	0.00	0.18	0.09
6	PDGFR	SFas	0	0	34475	0.45	0.27	0.45	0.00	0.18	0.09
7	BclxL	S1P	0	0	33922	0.45	0.27	0.45	0.00	0.18	0.09
8	MCL1	PDGFR	0	0	33606	0.45	0.27	0.45	0.00	0.18	0.09
9	IFNGT	S1P	1	0	33405	0.45	0.27	0.45	0.00	0.18	0.09
10	S1P	ZAP70	0	0	33168	0.45	0.27	0.45	0.00	0.18	0.09
11	IL2RB	S1P	0	0	32959	0.45	0.27	0.45	0.00	0.18	0.09
12	IL2RA	S1P	0	0	32580	0.45	0.27	0.45	0.00	0.18	0.09
13	FasL	PDGFR	1	0	31990	0.45	0.27	0.45	0.00	0.18	0.09
14	MCL1	SFas	0	0	31931	0.09	0.36	0.09	0.09	0.36	0.09
15	CREB	S1P	0	0	31818	0.45	0.27	0.45	0.00	0.18	0.09
16	PDGFR	TRADD	0	0	31488	0.45	0.27	0.45	0.00	0.18	0.09
17	IL2RBT	S1P	0	0	31236	0.45	0.27	0.45	0.00	0.18	0.09
18	S1P	SOCS	0	0	31202	0.45	0.27	0.45	0.00	0.18	0.09
19	S1P	TBET	0	1	31043	0.55	0.27	0.55	0.00	0.18	0.00
20	LCK	S1P	0	0	30996	0.45	0.27	0.45	0.00	0.18	0.09
21	BclxL	PDGFR	0	0	30821	0.45	0.27	0.45	0.00	0.18	0.09
22	NFKB	S1P	1	0	30689	0.45	0.27	0.45	0.00	0.18	0.09
23	FasL	SFas	1	0	30382	0.09	0.36	0.09	0.09	0.36	0.09
24	IFNGT	PDGFR	1	0	30344	0.45	0.27	0.45	0.00	0.18	0.09
25	GAP	S1P	0	0	30321	0.45	0.27	0.45	0.00	0.18	0.09
26	ERK	S1P	0	0	30080	0.45	0.27	0.45	0.00	0.18	0.09
27	PDGFR	ZAP70	0	0	30062	0.45	0.27	0.45	0.00	0.18	0.09
28	IL2RB	PDGFR	0	0	29864	0.45	0.27	0.45	0.00	0.18	0.09
29	SFas	TRADD	0	0	29762	0.09	0.36	0.09	0.09	0.36	0.09
30	FasL	MCL1	1	0	29544	0.09	0.36	0.09	0.09	0.36	0.09

Table 9: Top 30 2-Templates for Leukemia Network Basin 3

RANK	Variables		Values		FREQ	SUCCESS	B1	B2	B3	B4	B5
1	Ceramide	PDGFR	0	1	14404	0.45	0.27	0.00	0.45	0.27	0.00
2	Ceramide	S1P	0	1	14404	0.45	0.27	0.00	0.45	0.27	0.00
3	PDGFR	S1P	1	1	14404	0.64	0.18	0.00	0.64	0.18	0.00
4	Ceramide	IFNG	0	0	11323	0.09	0.36	0.09	0.09	0.36	0.09
5	IFNG	PDGFR	0	1	11323	0.09	0.45	0.00	0.09	0.45	0.00
6	IFNG	S1P	0	1	11323	0.45	0.27	0.00	0.45	0.27	0.00
7	Ceramide	PLCG1	0	1	11278	0.09	0.36	0.18	0.09	0.36	0.00
8	PDGFR	PLCG1	1	1	11278	0.09	0.45	0.00	0.09	0.45	0.00
9	PLCG1	S1P	1	1	11278	0.45	0.27	0.00	0.45	0.27	0.00
10	Ceramide	PI3K	0	1	11256	0.09	0.36	0.18	0.09	0.36	0.00
11	PDGFR	PI3K	1	1	11256	0.09	0.55	0.00	0.09	0.36	0.00
12	PI3K	S1P	1	1	11256	0.45	0.27	0.00	0.45	0.27	0.00
13	Ceramide	MCL1	0	0	11018	0.09	0.36	0.09	0.09	0.36	0.09
14	MCL1	PDGFR	0	1	11018	0.09	0.45	0.00	0.09	0.45	0.00
15	MCL1	S1P	0	1	11018	0.45	0.27	0.00	0.45	0.27	0.00
16	Ceramide	FasL	0	1	10664	0.09	0.36	0.09	0.09	0.36	0.09
17	FasL	PDGFR	1	1	10664	0.09	0.45	0.00	0.09	0.45	0.00
18	FasL	S1P	1	1	10664	0.45	0.27	0.00	0.45	0.27	0.00
19	Ceramide	TRADD	0	0	10376	0.09	0.36	0.09	0.09	0.36	0.09
20	PDGFR	TRADD	1	0	10376	0.09	0.45	0.00	0.09	0.45	0.00
21	S1P	TRADD	1	0	10376	0.45	0.27	0.00	0.45	0.27	0.00
22	BclxL	Ceramide	0	0	10289	0.09	0.36	0.09	0.09	0.36	0.09
23	BclxL	PDGFR	0	1	10289	0.09	0.45	0.00	0.09	0.45	0.00
24	BclxL	S1P	0	1	10289	0.45	0.27	0.00	0.45	0.27	0.00
25	Ceramide	IFNGT	0	1	10043	0.09	0.36	0.09	0.09	0.36	0.09
26	IFNGT	PDGFR	1	1	10043	0.09	0.45	0.00	0.09	0.45	0.00
27	IFNGT	S1P	1	1	10043	0.45	0.27	0.00	0.45	0.27	0.00
28	Ceramide	IL2RA	0	0	9994	0.09	0.36	0.09	0.09	0.36	0.09
29	IL2RA	PDGFR	0	1	9994	0.09	0.45	0.00	0.09	0.45	0.00
30	IL2RA	S1P	0	1	9994	0.45	0.27	0.00	0.45	0.27	0.00

Table 10: Top 30 2-Templates for Leukemia Network Basin 4

RANK	Variables		Values		FREQ	SUCCESS	B1	B2	B3	B4	B5
1	FasL	NFKB	1	1	6105	0.36	0.36	0.09	0.09	0.36	0.09
2	MCL1	NFKB	0	1	5885	0.36	0.36	0.09	0.09	0.36	0.09
3	NFKB	TRADD	1	0	5766	0.36	0.36	0.09	0.09	0.36	0.09
4	NFKB	PI3K	1	1	5653	0.36	0.36	0.18	0.09	0.36	0.00
5	IL2RBT	NFKB	0	1	5625	0.36	0.36	0.09	0.09	0.36	0.09
6	FasT	NFKB	1	1	5618	0.36	0.36	0.09	0.09	0.36	0.09
7	FasL	MCL1	1	0	5617	0.36	0.36	0.09	0.09	0.36	0.09
8	FasL	TRADD	1	0	5509	0.36	0.36	0.09	0.09	0.36	0.09
9	MCL1	TRADD	0	0	5463	0.36	0.36	0.09	0.09	0.36	0.09
10	NFKB	SOCS	1	0	5454	0.36	0.36	0.09	0.09	0.36	0.09
11	FasL	FasT	1	1	5391	0.36	0.36	0.09	0.09	0.36	0.09
12	FasL	IL2RBT	1	0	5367	0.36	0.36	0.09	0.09	0.36	0.09
13	BclxL	NFKB	0	1	5336	0.36	0.36	0.09	0.09	0.36	0.09
14	IL2RB	NFKB	0	1	5333	0.36	0.36	0.09	0.09	0.36	0.09
15	FasL	PI3K	1	1	5326	0.36	0.36	0.18	0.09	0.36	0.00
16	IL2RBT	MCL1	0	0	5307	0.36	0.36	0.09	0.09	0.36	0.09
17	JAK	TBET	0	0	5306	0.73	0.00	0.00	0.09	0.73	0.18
18	NFKB	ZAP70	1	0	5282	0.36	0.36	0.09	0.09	0.36	0.09
19	NFKB	TNF	1	1	5258	0.36	0.36	0.09	0.09	0.36	0.09
20	FasT	TNF	1	1	5247	0.36	0.36	0.09	0.09	0.36	0.09
21	NFKB	TBET	1	0	5230	0.73	0.00	0.00	0.09	0.73	0.18
22	CREB	NFKB	0	1	5221	0.36	0.36	0.09	0.09	0.36	0.09
23	MCL1	PI3K	0	1	5201	0.36	0.36	0.18	0.09	0.36	0.00
24	IFNG	NFKB	0	1	5179	0.36	0.36	0.09	0.09	0.36	0.09
25	NFKB	PLCG1	1	1	5168	0.36	0.36	0.18	0.09	0.36	0.00
26	FasL	SOCS	1	0	5155	0.36	0.36	0.09	0.09	0.36	0.09
27	FasL	TNF	1	1	5149	0.36	0.36	0.09	0.09	0.36	0.09
28	JAK	NFKB	0	1	5144	0.36	0.36	0.09	0.09	0.36	0.09
29	IL2RBT	TRADD	0	0	5143	0.36	0.36	0.09	0.09	0.36	0.09
30	FasT	TRADD	1	0	5139	0.36	0.36	0.09	0.09	0.36	0.09

Table 11: Top 30 3-Templates for Leukemia Network Basin 1

RANK		Variables			Values		FREQ	SUCCESS	B1	B2	B3	B4	B5
1	FasL	IFNGT	MCL1	1	1	0	24553	0.36	0.36	0.09	0.09	0.36	0.09
2	FasL	MCL1	TRADD	1	0	0	23890	0.36	0.36	0.09	0.09	0.36	0.09
3	FasL	IFNG	MCL1	1	0	0	23812	0.36	0.36	0.09	0.09	0.36	0.09
4	Ceramide	FasL	MCL1	0	1	0	23772	0.36	0.36	0.09	0.09	0.36	0.09
5	BclxL	FasL	MCL1	0	1	0	23607	0.36	0.36	0.09	0.09	0.36	0.09
6	Ceramide	IFNG	MCL1	0	0	0	23494	0.36	0.36	0.09	0.09	0.36	0.09
7	FasL	MCL1	TBET	1	0	1	23484	0.36	0.36	0.18	0.09	0.36	0.00
8	BclxL	IFNG	MCL1	0	0	0	23482	0.36	0.36	0.09	0.09	0.36	0.09
9	IFNGT	MCL1	TBET	1	0	1	23257	0.36	0.36	0.18	0.09	0.36	0.00
10	IFNG	IFNGT	MCL1	0	1	0	23234	0.36	0.36	0.09	0.09	0.36	0.09
11	FasL	IL2RA	MCL1	1	0	0	23113	0.36	0.36	0.09	0.09	0.36	0.09
12	FasL	IFNGT	TBET	1	1	1	23044	0.36	0.36	0.18	0.09	0.36	0.00
13	FasL	IFNGT	TRADD	1	1	0	23018	0.36	0.36	0.09	0.09	0.36	0.09
14	BclxL	Ceramide	MCL1	0	0	0	22998	0.36	0.36	0.09	0.09	0.36	0.09
15	FasL	IFNG	IFNGT	1	0	1	22970	0.36	0.36	0.09	0.09	0.36	0.09
16	IFNG	IL2RA	MCL1	0	0	0	22950	0.36	0.36	0.09	0.09	0.36	0.09
17	BclxL	IFNGT	MCL1	0	1	0	22937	0.36	0.36	0.09	0.09	0.36	0.09
18	Ceramide	PDGFR	S1P	0	1	0	22936	0.73	0.73	0.00	0.00	0.27	0.00
19	Fas	FasL	MCL1	0	1	0	22860	0.55	0.55	0.09	0.09	0.18	0.09
20	Ceramide	FasL	IFNGT	0	1	1	22851	0.36	0.36	0.09	0.09	0.36	0.09
21	Ceramide	MCL1	TRADD	0	0	0	22838	0.36	0.36	0.09	0.09	0.36	0.09
22	IFNG	MCL1	TRADD	0	0	0	22825	0.36	0.36	0.09	0.09	0.36	0.09
23	Ceramide	IFNGT	MCL1	0	1	0	22820	0.36	0.36	0.09	0.09	0.36	0.09
24	BclxL	MCL1	TRADD	0	0	0	22809	0.36	0.36	0.09	0.09	0.36	0.09
25	IFNGT	MCL1	TRADD	1	0	0	22784	0.36	0.36	0.09	0.09	0.36	0.09
26	BclxL	MCL1	TBET	0	0	1	22735	0.36	0.36	0.18	0.09	0.36	0.00
27	IFNG	MCL1	TBET	0	0	1	22684	0.36	0.36	0.18	0.09	0.36	0.00
28	BclxL	IL2RA	MCL1	0	0	0	22651	0.36	0.36	0.09	0.09	0.36	0.09
29	Ceramide	MCL1	TBET	0	0	1	22631	0.36	0.36	0.18	0.09	0.36	0.00
30	Ceramide	FasL	IFNG	0	1	0	22599	0.36	0.36	0.09	0.09	0.36	0.09

Table 12: Top 30 3-Templates for Leukemia Network Basin 2

RANK		Variables			Values		FREQ	SUCCESS	B1	B2	B3	B4	B5
1	PDGFR	S1P	SFas	0	0	0	34475	0.91	0.00	0.91	0.00	0.00	0.09
2	MCL1	PDGFR	S1P	0	0	0	33606	0.91	0.00	0.91	0.00	0.00	0.09
3	FasL	PDGFR	S1P	1	0	0	31990	0.91	0.00	0.91	0.00	0.00	0.09
4	MCL1	S1P	SFas	0	0	0	31931	0.45	0.27	0.45	0.00	0.18	0.09
5	PDGFR	S1P	TRADD	0	0	0	31488	0.91	0.00	0.91	0.00	0.00	0.09
6	BclxL	PDGFR	S1P	0	0	0	30821	0.91	0.00	0.91	0.00	0.00	0.09
7	MCL1	PDGFR	SFas	0	0	0	30409	0.45	0.27	0.45	0.00	0.18	0.09
8	FasL	S1P	SFas	1	0	0	30382	0.45	0.27	0.45	0.00	0.18	0.09
9	IFNGT	PDGFR	S1P	1	0	0	30344	0.91	0.00	0.91	0.00	0.00	0.09
10	PDGFR	S1P	ZAP70	0	0	0	30062	0.91	0.00	0.91	0.00	0.00	0.09
11	IL2RB	PDGFR	S1P	0	0	0	29864	0.91	0.00	0.91	0.00	0.00	0.09
12	S1P	SFas	TRADD	0	0	0	29762	0.45	0.27	0.45	0.00	0.18	0.09
13	FasL	MCL1	S1P	1	0	0	29544	0.45	0.27	0.45	0.00	0.18	0.09
14	IL2RA	PDGFR	S1P	0	0	0	29501	0.91	0.00	0.91	0.00	0.00	0.09
15	BclxL	S1P	SFas	0	0	0	29136	0.45	0.27	0.45	0.00	0.18	0.09
16	MCL1	S1P	TRADD	0	0	0	28953	0.45	0.27	0.45	0.00	0.18	0.09
17	FasL	PDGFR	SFas	1	0	0	28859	0.45	0.27	0.45	0.00	0.18	0.09
18	CREB	PDGFR	S1P	0	0	0	28723	0.91	0.00	0.91	0.00	0.00	0.09
19	IFNGT	S1P	SFas	1	0	0	28720	0.45	0.27	0.45	0.00	0.18	0.09
20	BclxL	MCL1	S1P	0	0	0	28564	0.45	0.27	0.45	0.00	0.18	0.09
21	S1P	SFas	ZAP70	0	0	0	28382	0.45	0.27	0.45	0.00	0.18	0.09
22	PDGFR	SFas	TRADD	0	0	0	28250	0.45	0.27	0.45	0.00	0.18	0.09
23	IL2RB	S1P	SFas	0	0	0	28227	0.45	0.27	0.45	0.00	0.18	0.09
24	IL2RBT	PDGFR	S1P	0	0	0	28104	0.91	0.00	0.91	0.00	0.00	0.09
25	FasL	S1P	TRADD	1	0	0	28090	0.45	0.27	0.45	0.00	0.18	0.09
26	PDGFR	S1P	SOCS	0	0	0	28072	0.91	0.00	0.91	0.00	0.00	0.09
27	PDGFR	S1P	TBET	0	0	1	28057	1.00	0.00	1.00	0.00	0.00	0.00
28	FasL	MCL1	PDGFR	1	0	0	28007	0.45	0.27	0.45	0.00	0.18	0.09
29	LCK	PDGFR	S1P	0	0	0	27944	0.91	0.00	0.91	0.00	0.00	0.09
30	IL2RA	S1P	SFas	0	0	0	27889	0.45	0.27	0.45	0.00	0.18	0.09

Table 13: Top 30 3-Templates for Leukemia Network Basin 3

RANK	Variables			Values			FREQ	SUCCESS	B1	B2	B3	B4	B5
1	Ceramide	PDGFR	S1P	0	1	1	14404	1.00	0.00	0.00	1.00	0.00	0.00
2	Ceramide	IFNG	PDGFR	0	0	1	11323	0.45	0.27	0.00	0.45	0.27	0.00
3	Ceramide	IFNG	S1P	0	0	1	11323	0.45	0.27	0.00	0.45	0.27	0.00
4	IFNG	PDGFR	S1P	0	1	1	11323	0.64	0.18	0.00	0.64	0.18	0.00
5	Ceramide	PDGFR	PLCG1	0	1	1	11278	0.45	0.27	0.00	0.45	0.27	0.00
6	Ceramide	PLCG1	S1P	0	1	1	11278	0.45	0.27	0.00	0.45	0.27	0.00
7	PDGFR	PLCG1	S1P	1	1	1	11278	0.64	0.18	0.00	0.64	0.18	0.00
8	Ceramide	PDGFR	PI3K	0	1	1	11256	0.45	0.36	0.00	0.45	0.18	0.00
9	Ceramide	PI3K	S1P	0	1	1	11256	0.45	0.27	0.00	0.45	0.27	0.00
10	PDGFR	PI3K	S1P	1	1	1	11256	0.64	0.18	0.00	0.64	0.18	0.00
11	Ceramide	MCL1	PDGFR	0	0	1	11018	0.45	0.27	0.00	0.45	0.27	0.00
12	Ceramide	MCL1	S1P	0	0	1	11018	0.45	0.27	0.00	0.45	0.27	0.00
13	MCL1	PDGFR	S1P	0	1	1	11018	0.64	0.18	0.00	0.64	0.18	0.00
14	Ceramide	FasL	PDGFR	0	1	1	10664	0.45	0.27	0.00	0.45	0.27	0.00
15	Ceramide	FasL	S1P	0	1	1	10664	0.45	0.27	0.00	0.45	0.27	0.00
16	FasL	PDGFR	S1P	1	1	1	10664	0.64	0.18	0.00	0.64	0.18	0.00
17	Ceramide	PDGFR	TRADD	0	1	0	10376	0.45	0.27	0.00	0.45	0.27	0.00
18	Ceramide	S1P	TRADD	0	1	0	10376	0.45	0.27	0.00	0.45	0.27	0.00
19	PDGFR	S1P	TRADD	1	1	0	10376	0.64	0.18	0.00	0.64	0.18	0.00
20	BclxL	Ceramide	PDGFR	0	0	1	10289	0.45	0.27	0.00	0.45	0.27	0.00
21	BclxL	Ceramide	S1P	0	0	1	10289	0.45	0.27	0.00	0.45	0.27	0.00
22	BclxL	PDGFR	S1P	0	1	1	10289	0.64	0.18	0.00	0.64	0.18	0.00
23	Ceramide	IFNGT	PDGFR	0	1	1	10043	0.45	0.27	0.00	0.45	0.27	0.00
24	Ceramide	IFNGT	S1P	0	1	1	10043	0.45	0.27	0.00	0.45	0.27	0.00
25	IFNGT	PDGFR	S1P	1	1	1	10043	0.64	0.18	0.00	0.64	0.18	0.00
26	Ceramide	IL2RA	PDGFR	0	0	1	9994	0.45	0.27	0.00	0.45	0.27	0.00
27	Ceramide	IL2RA	S1P	0	0	1	9994	0.45	0.27	0.00	0.45	0.27	0.00
28	IL2RA	PDGFR	S1P	0	1	1	9994	0.64	0.18	0.00	0.64	0.18	0.00
29	Ceramide	DISC	PDGFR	0	0	1	9927	0.45	0.27	0.00	0.45	0.27	0.00
30	Ceramide	DISC	S1P	0	0	1	9927	0.45	0.45	0.00	0.45	0.09	0.00

Table 14: Top 30 3-Templates for Leukemia Network Basin 4

RANK	Variables			Values			FREQ	SUCCESS	B1	B2	B3	B4	B5
1	FasL	MCL1	NFKB	1	0	1	5203	0.36	0.36	0.09	0.09	0.36	0.09
2	FasL	NFKB	TRADD	1	1	0	5147	0.36	0.36	0.09	0.09	0.36	0.09
3	MCL1	NFKB	TRADD	0	1	0	5051	0.36	0.36	0.09	0.09	0.36	0.09
4	FasL	FasT	NFKB	1	1	1	5031	0.36	0.36	0.09	0.09	0.36	0.09
5	FasL	IL2RBT	NFKB	1	0	1	4954	0.36	0.36	0.09	0.09	0.36	0.09
6	FasL	NFKB	SOCS	1	1	0	4938	0.36	0.36	0.09	0.09	0.36	0.09
7	FasL	MCL1	TRADD	1	0	0	4920	0.36	0.36	0.09	0.09	0.36	0.09
8	FasL	NFKB	PI3K	1	1	1	4904	0.36	0.36	0.18	0.09	0.36	0.00
9	FasT	NFKB	TNF	1	1	1	4869	0.36	0.36	0.09	0.09	0.36	0.09
10	MCL1	NFKB	SOCS	0	1	0	4852	0.36	0.36	0.09	0.09	0.36	0.09
11	IL2RBT	MCL1	NFKB	0	0	1	4848	0.36	0.36	0.09	0.09	0.36	0.09
12	FasL	FasT	TNF	1	1	1	4823	0.36	0.36	0.09	0.09	0.36	0.09
13	FasT	NFKB	TRADD	1	1	0	4779	0.36	0.36	0.09	0.09	0.36	0.09
14	FasL	NFKB	TNF	1	1	1	4764	0.36	0.36	0.09	0.09	0.36	0.09
15	MCL1	NFKB	PI3K	0	1	1	4725	0.36	0.36	0.18	0.09	0.36	0.00
16	NFKB	SOCS	TRADD	1	0	0	4720	0.36	0.36	0.09	0.09	0.36	0.09
17	FasL	FasT	TRADD	1	1	0	4718	0.36	0.36	0.09	0.09	0.36	0.09
18	IL2RBT	NFKB	TRADD	0	1	0	4712	0.36	0.36	0.09	0.09	0.36	0.09
19	FasL	IL2RBT	MCL1	1	0	0	4691	0.36	0.36	0.09	0.09	0.36	0.09
20	NFKB	PI3K	TRADD	1	1	0	4683	0.36	0.36	0.18	0.09	0.36	0.00
21	FasL	MCL1	SOCS	1	0	0	4682	0.36	0.36	0.09	0.09	0.36	0.09
22	FasT	TNF	TRADD	1	1	0	4677	0.36	0.36	0.09	0.09	0.36	0.09
23	FasL	IL2RB	NFKB	1	0	1	4658	0.36	0.36	0.09	0.09	0.36	0.09
24	BclxL	FasL	NFKB	0	1	1	4638	0.36	0.36	0.09	0.09	0.36	0.09
25	IL2RBT	NFKB	SOCS	0	1	0	4632	0.36	0.36	0.09	0.09	0.36	0.09
26	FasL	NFKB	ZAP70	1	1	0	4621	0.36	0.36	0.09	0.09	0.36	0.09
27	FasT	MCL1	NFKB	1	0	1	4619	0.36	0.36	0.09	0.09	0.36	0.09
28	IL2RB	MCL1	NFKB	0	0	1	4612	0.36	0.36	0.09	0.09	0.36	0.09
29	NFKB	TNF	TRADD	1	1	0	4612	0.36	0.36	0.09	0.09	0.36	0.09
30	FasL	SOCS	TRADD	1	0	0	4600	0.36	0.36	0.09	0.09	0.36	0.09

References

[1] Assieh Saadatpour, István Albert, and Réka Albert. Attractor analysis of asynchronous boolean models of signal transduction networks. *Journal of theoretical biology*, 266(4):641–656, October 2010.

[2] Assieh Saadatpour, Rui-Sheng Wang, Aijun Liao, Xin Liu, Thomas P. Loughran, István Albert, and Réka Albert. Dynamical and structural analysis of a t cell survival network identifies novel candidate therapeutic targets for large granular lymphocyte leukemia. *PLoS Comput Biol*, 7(11):e1002267+, November 2011.

[3] Ranran Zhang, Mithun V. Shah, Jun Yang, Susan B. Nyland, Xin Liu, Jong K. Yun, Réka Albert, and Thomas P. Loughran. Network model of survival signaling in large granular lymphocyte leukemia. *Proceedings of the National Academy of Sciences*, 105(42):16308–16313, October 2008.
