# Supplementary figures and images for: Template-based intervention in Boolean network models of biological systems
Source: EURASIP J Bioinform Syst Biol. 2014 Jul 19;2014:11. doi: 10.1186/s13637-014-0011-4 (PMC5270454; doi:10.1186/s13637-014-0011-4)

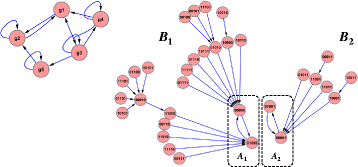

Supplement: Supplementary file 4 — Authors’ original file for figure 1 [file 13637_2014_11_MOESM4_ESM.gif]

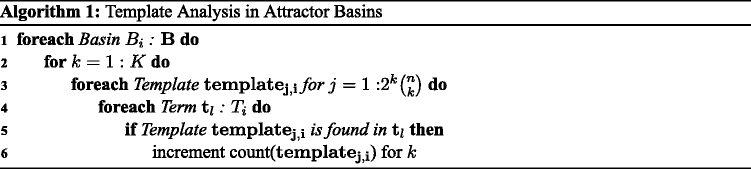

Supplement: Supplementary file 5 — Authors’ original file for figure 2 [file 13637_2014_11_MOESM5_ESM.gif]

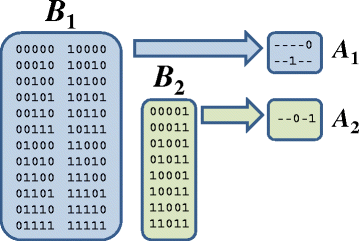

Supplement: Supplementary file 6 — Authors’ original file for figure 3 [file 13637_2014_11_MOESM6_ESM.gif]

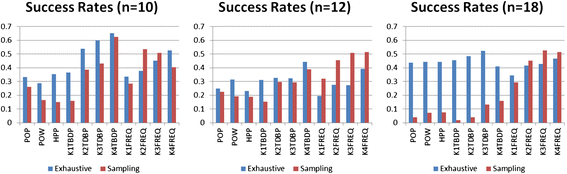

Supplement: Supplementary file 7 — Authors’ original file for figure 4 [file 13637_2014_11_MOESM7_ESM.gif]

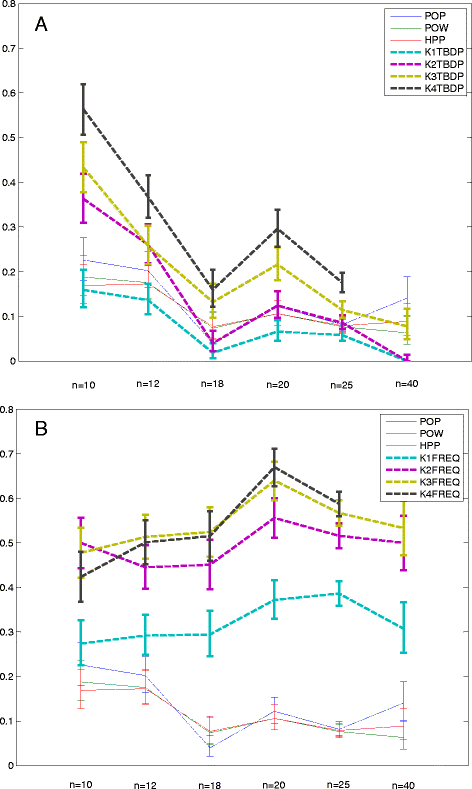

Supplement: Supplementary file 8 — Authors’ original file for figure 5 [file 13637_2014_11_MOESM8_ESM.gif]

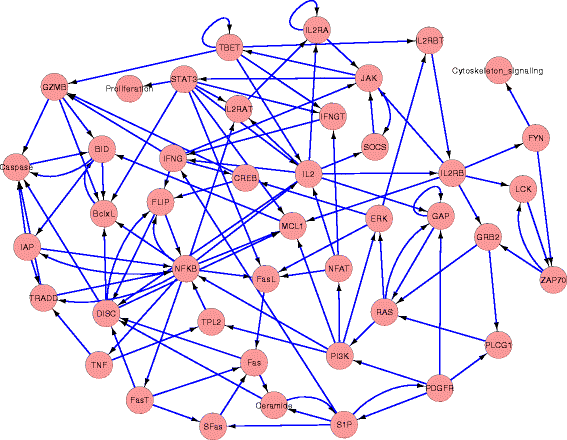

Supplement: Supplementary file 9 — Authors’ original file for figure 6 [file 13637_2014_11_MOESM9_ESM.gif]
